# Evidence and gap map report: Social and Behavior Change Communication (SBCC) interventions for strengthening HIV prevention and research among adolescent girls and young women (AGYW) in low‐ and middle‐income countries (LMICs)

**DOI:** 10.1002/cl2.1297

**Published:** 2023-01-10

**Authors:** Devi Leena Bose, Anhad Hundal, Sabina Singh, Shweta Singh, Kuhika Seth, Saif ul Hadi, Ashrita Saran, Jessy Joseph, Kriti Goyal, Solomon Salve

**Affiliations:** ^1^ IAVI New Delhi India; ^2^ Campbell South Asia New Delhi India

## Abstract

**Background:**

Adolescent girls and young women (AGYW), aged 15–24 years, are disproportionately affected by HIV and other sexual and reproductive health (SRH) risks due to varying social, cultural, and economic factors that affect their choices and shape their knowledge, understanding, and practices with regard to their health. Socio‐Behavioral Change Communication (SBCC) interventions targeted at strengthening the capabilities of individuals and their networks have supported the demand and uptake of prevention services and participation in biomedical research. However, despite growing global recognition of the domain, high‐quality evidence on the effectiveness of SBCC remains scattered. This evidence and gap map (EGM) report characterizes the evidence base on SBCC interventions for strengthening HIV Prevention and Research among AGYW in low‐ and middle‐income countries (LMICs), identifying evidence gaps and outlining the scope of future research and program design.

**Objectives:**

The objectives of the proposed EGM are to: (a) identify and map existing EGMs in the use of diverse SBCC strategies to strengthen the adoption of HIV prevention measures and participation in research among AGYW in LMICs and (b) identify areas where more interventions and evidence are needed to inform the design of future SBCC strategies and programs for AGYW engagement in HIV prevention and research.

**Methods:**

This EGM is based on a comprehensive search of systematic reviews and impact evaluations corresponding to a range of interventions and outcomes—aimed at engaging AGYW in HIV prevention and research ‐ that were published in LMICs from January 2000 to April 2021. Based on guidance for producing a Campbell Collaboration EGM, the intervention and outcome framework was designed in consultation with a group of experts. These interventions were categorized across four broad intervention themes: mass‐media, community‐based, interpersonal, and Information Communication and Technology (ICT)/Digital Media‐based interventions. They were further sub‐categorized into 15 intervention categories. Included studies looked at 23 unique behavioral and health outcomes such as knowledge attitude and skills, relationship dynamics, household dynamics, health care services, and health outcomes and research engagement. The EGM is presented as a matrix in which the rows are intervention categories/sub‐categories, and the columns are outcome domains/subdomains. Each cell is mapped to an intervention targeted at outcomes. Additional filters like region, country, study design, age group, funding agency, influencers, population group, publication status, study confidence, setting, and year of publication have been added.

**Selection Criteria:**

To be eligible, studies must have tested the effectiveness of SBCC interventions at engaging AGYW in LMICs in HIV prevention and research. The study sample must have consisted of AGYW between the ages of 15–24, as defined by UNAIDS. Both experimental (random assignment) and quasi‐experimental studies that included a comparison group were eligible. Relevant outcomes included those at the individual, influencer, and institutional levels, along with those targeting research engagement and prevention‐related outcomes.

**Results:**

This EGM comprises 415 impact evaluations and 43 systematic reviews. Interventions like peer‐led interactions, counseling, and community dialogues were the most dominant intervention sub‐types. Despite increased digital penetration use of media and technology‐driven interventions are relatively less studied. Most of the interventions were delivered by peers, health care providers, and educators, largely in school‐based settings, and in many cases are part of sex‐education curricula. Evidence across geographies was mostly concentrated in Sub‐Saharan Africa (70%). Most measured outcomes focused on disease‐related knowledge dissemination and enhancing awareness of available prevention options/strategies. These included messaging around consistent condom use, limiting sexual partners, routine testing, and awareness. Very few studies were able to include psychographic, social, and contextual factors influencing AGYW health behaviors and decisions, especially those measuring the impact of social and gender norms, relationship dynamics, and household dynamics‐related outcomes. Outcomes related to engagement in the research were least studied.

**Conclusion:**

This EGM highlights that evidence is heavily concentrated within the awareness‐intent spectrum of behavior change and gets lean for outcomes situated within the intent‐action and the action‐habit formation spectrum of the behavior change continuum. Most of the evidence was concentrated on increasing awareness, knowledge, and building risk perception around SRH domains, however, fewer studies focused on strengthening the agency and self‐efficacy of individuals. Similarly, evidence on extrinsic factors—such as strengthening social and community norms, relationships, and household dynamics—that determine individual thought and action such as negotiation and life skills were also found to be less populated. Few studies explore the effectiveness of these interventions across diverse AGYW identities, like pregnant women and new mothers, sex workers, and people living with HIV, leading to limited understanding of the use of these interventions across multiple user segments including key influencers such as young men, partners, families, religious leaders, and community elders was relatively low. There is a need for better quality evidence that accounts for the diversity of experiences within these populations to understand what interventions work, for whom, and toward what outcome. Further, the evidence for use of digital and mass‐media tools remains poorly populated. Given the increasing penetration of these tools and growing media literacy on one end, with widening gender‐based gaps on the other, it is imperative to gather more high‐quality evidence on their effectiveness. Timely evidence generation can help leverage these platforms appropriately and enable intervention designs that are responsive to changing communication ecologies of AGYW. SBCC can play a critical role in helping researchers meaningfully engage and collaborate with communities as equal stakeholders, however, this remains poorly evidenced and calls for investigation and investment. A full list of abbreviations and acronyms are available in Supporting Information: Appendix F.

## 1 PLAIN LANGUAGE SUMMARY

High‐quality evidence on the effectiveness of SBCC interventions for strengthening HIV prevention and research among adolescent girls and young women (AGYW) remains scattered and poorly documented, despite growing global recognition of the domain.

### 1.1 Background

AGYW, aged 15–24 years, are disproportionately affected by HIV and other sexual and reproductive health (SRH). Converging social, cultural, and economic factors affect how adolescent girls and young women understand, negotiate and access information and biomedical treatment related to HIV. SBCC interventions form a pivotal part of the comprehensive HIV prevention response as they not only help address aspects of underlying socio‐behavioral barriers that drive the epidemic, but also play a critical role in expanding knowledge of, and access to, quality health services, health equity and participation in research. They also assist in addressing gendered health disparities throughout the continuum of care (CDC, 2017). While the global buy‐in for socio‐behavioral interventions to support demand and uptake of HIV services is on the rise, evidence and impact of this in the context of AGYW needs to be systematically outlined and enumerated to enable informed decision‐making by sponsors, researchers, policymakers and public health experts working on HIV.

### 1.2 What is the Evidence and Gap Map about?

AGYW around the world faces serious and multi‐pronged challenges to achieving their sexual and reproductive health rights, including vulnerability to HIV and STIs. To this end, evidence‐informed interventions for HIV prevention, care, and uptake targeting AGYW are critical in addressing these challenges and identifying gaps in programming. This EGM presents a consolidated evidence base of existing SBCC interventions, and the AGYW end‐user behaviors they target, in low‐and‐middle‐income countries. It looks at a range of SBCC strategies including those employing mass media, community‐based interventions, interpersonal interventions, and ICT and digital media‐based interventions.

1 Aim of the EGM

The aim of this EGM is to identify, map, and present all the available evidence from systematic reviews and impact evaluations of interventions to strengthen the adoption of HIV prevention measures and participation in research among AGYW in LMICs.

### 1.3 What studies are included?

The EGM includes 458 studies: 43 systematic reviews and 415 impact evaluations, published in the English language from 2000 onwards.

### 1.4 What is the distribution of evidence?

Evidence remains unevenly distributed across intervention and outcome categories. The most populated interventions include interpersonal communication, followed by community‐based interventions. Most of these interventions were delivered by health care providers and educators. Further, evidence across geographies was mostly concentrated in Sub‐Saharan Africa.

Most measured outcomes focused on disease‐related knowledge dissemination and enhancing awareness of available prevention options/strategies. These included messaging around consistent condom use, limiting sexual partners, routine testing, and awareness. Very few studies included psychographic, social, and contextual factors influencing AGYW health behaviors and decisions, especially those measuring the impact of social and gender norms, relationship dynamics, and household dynamics‐related outcomes. Outcomes related to engagement in research were least studied.

### 1.5 What do the findings of this map mean?

Behavior change is a dynamic process and is influenced by multiple factors that constantly interact with each other across individual, social and structural levels. Individual behavior is shaped by varying factors that affect their capabilities to make decisions, enable them to translate intent into action and motivate them to make long‐term behavioral shifts (Michie et al., 2014).

Most studies in this EGM focus on enhancing the knowledge‐related capabilities to create behavioral and health impacts situated within the awareness‐intent spectrum of behavior change. However, evidence for interventions targeting outcomes situated within the intent‐action and the action‐habit formation spectrum are poorly populated. This highlights the need for more studies to focus on interventions that help achieve envisioned behavioral and health outcomes across the continuum that include various stages—awareness, knowledge, intent, trial, action, and habit formation (Prochaska & DiClemente, 1983).

Further, very few studies explore the effectiveness of these interventions across diverse AGYW identities—such as pregnant women and new mothers (PWNM), sex workers, and people living with HIV (PLHIV)—leading to a limited understanding of the use of these interventions across critical sub‐populations and multiple user segments. This underscores the need for better quality evidence to understand what interventions work, for whom, and towards what outcome. It is, therefore, important to account for the diversity of experiences within AGYW populations, and better understand behavioral convergences and divergences to inform intervention design and evidence generation.

Realizing the value of communication channels in shaping behavior change discourse, this EGM actively looks at evidence across different communication strategies and platforms. However, we found that evidence towards the use of digital media tools such as social media and mobile‐based services remains poorly populated. Studies on the use of popular culture tools—including mass‐media, theatre, and arts‐based approaches—are also relatively low. Given the penetration of digital tools, increased access to mass media and growing media literacy on one end, with ever‐widening gender‐based gaps on the other, it is imperative to gather more high‐quality evidence on the effectiveness of these tools. Timely evidence generation can help leverage this medium appropriately and enable intervention designs that are responsive to the changing communication ecologies of AGYW.

### 1.6 How up to date is this EGM?

The authors searched for studies published up to April 2021.

### 1.7 Positionality and Ethics Statement

The lead author, Devi Leena Bose, along with Anhad Hundal and Saif ul Hadi position ourselves as SBCC practitioners from LMIC working towards mainstreaming SBCC practices as an approach to help achieve sustainable development goals. We have been working with IAVI a nonprofit scientific research organization that develops vaccines and antibodies for HIV, tuberculosis, emerging infectious diseases (including COVID‐19), and neglected diseases. Through its various programs spread across India and Africa, IAVI aims to use SBCC strategies towards increasing knowledge, improving attitudes and enhancing practices of individuals and communities towards positive health seeking behavior and reduce barriers of vulnerable populations to contribute more to research and access prevention services. IAVI has co‐created evidence‐driven SBCC interventions for enhancing science literacy, research acceptance, research participation, using human‐centered design and experiential learning. However, these interventions are not evaluated or influence the EGM in any manner. Other co‐authors have no conflict of interest and have contributed to the EGM from a technical and methdolodological perspective.

## 2 BACKGROUND

### 2.1 Introduction to the problem

Adolescent girls and young women (AGYW) are disproportionately affected by HIV and other sexual and reproductive health risks. Converging social, cultural, and economic factors affect how AGYW understand, negotiate, and access information and biomedical treatment related to HIV. Persistent gender and age disparities and stigmas around female sexuality faced by this group in the impact of HIV are both exacerbated by, and reinforce, issues such as poverty, lack of access to education (including sexual and reproductive health education) and livelihood opportunities, limited financial autonomy, lack of access to sexual and reproductive healthcare (as well as other healthcare services), and the risk of violence, including intimate partner violence (IPV). A UNAIDS report found that nearly 1 in 3 women globally has experienced physical and/or sexual violence by an intimate partner, non‐partner, or both, in their lifetimes (World Health Organization, 2021). Early marriage also poses special risks to young people, particularly women.

For this reason, many organizations have put AGYW at the center of their response to HIV, including UNAIDS (2016), UNFPA (2016), WHO (2017), and national bodies like the National AIDS Control Organization (NACO) (2017) of India. According to UNAIDS (2019), 6200 women and adolescent girls between the ages of 15–24 years are newly infected with HIV every week, and 50 adolescent girls die from HIV‐related diseases every day, including treatable diseases like cervical cancer (Stegling, 2019). In 1995, 1.2 million adolescent girls and women above the age of 15 years acquired AIDS, consisting of half of all new infections globally. This number dropped by 39% in 2018, yet AGYW still accounted for 47% of new infections, falling short of the 2020 goal set by UNAIDS (UNAIDS, 2019). Further, in 2019, young people (15–49 years) accounted for an estimated 28% of all new infections globally, and the number of AGYW who acquired HIV (280,000) was nearly three times that of the fast‐track target for 2020.

As a signatory to the United Nations Declaration on Sustainable Development Goals (SDGs), India is committed to achieving the “end of the HIV/AIDS epidemic” as a public health threat by 2030 (UNAIDS, 2016). While the country has made immense strides in reducing HIV prevalence, with a 66% reduction in new infections since 2000 and a 54% reduction in AIDS‐related deaths since 2007 (NACO, 2017), there are still 2.3 million people living with HIV (NACO, 2017). Women constitute 44% of this population.

The National Family Health Survey of India (NFHS‐5), 2019–2021 (IIPS & MoHFW, 2021), highlights gender‐based gaps in awareness levels and health‐seeking behaviors—only 29% of women (defined as being between the ages of 15–49 years) reported having comprehensive knowledge about HIV, a marginal increase from the previous surveys 21%. It is important to note that, while the previous IIPS & MoHFW (2017) approximated that 44% of women knew where to get tested, the most recent survey has dropped HIV testing as a key indicator. Further, the survey found that, while there has been an increase in the current use of family planning methods amongst married women (69% as per the NFHS‐5, as compared to 54% reported in the NFHS‐4), female sterilization remained the most reported method used, followed by condoms. This highlights the need to closely examine behavioral differences in seeking healthcare between married and unmarried women, along with disaggregation's based on age, regional and other socio‐cultural aspects.

### 2.2 Intervention

Methodologies centered on behavioral change have proven to be effective in the adoption and adherence of better health‐seeking practices. While the global buy‐in for socio‐behavioral interventions to support demand and uptake of HIV services is on the rise, evidence mapping of the scope and impact of this in the context of AGYW needs to be systematically outlined and enumerated to help further adoption by researchers, policymakers and HIV program managers.

Social and Behavior Change Communication (SBCC) interventions form a pivotal part of the comprehensive HIV prevention response, as they not only help address aspects of underlying behavioral and social barriers that drive the epidemic, but also play a critical role in expanding knowledge of, and access to, quality health services, strengthen equity and participation and assist in addressing gendered health disparities throughout the continuum of care (CDC, 2017), thereby creating an enabling environment. However, there is also an understanding that, over the years, health communication has remained underutilized; not only is health communication often included in programs as an afterthought rather than integrated right in the beginning, the evidence towards creating this enabling environment, whereby uptake and support of prevention options and research is key, is often scattered (Sugg, 2016).

### 2.3 Why is it important to develop this evidence and gap map (EGM)?

This EGM is important to map and consolidate existing evidence on SBCC strategies for HIV prevention and research among AGYW in LMICs. More simply put, this map helps understand what kind of SBCC interventions have been used with which population groups, towards what behavioral and health outcomes and, further, helps identify areas where more evidence and interventions are needed.

The knowledge generated through this EGM has the potential to support numerous stakeholders, including intervention designers, policymakers, program managers, community engagement practitioners, socio‐behavioral researchers, donors, and the general public:

- For socio‐behavioral researchers, this exercise may provide an opportunity to explore the existing evidence base, be better informed about critical research gaps, and, therefore, design future research to address the same.
- For public health specialists and intervention designers, this EGM may provide a better understanding of the most effective strategies/approaches to engage diverse AGYW populations.
- For biomedical/public health researchers, this map may provide a preliminary understanding of an array of interventions and outcomes that can inform their methodologies to engage AGYW communities in research.
- For funders and decision‐makers, this EGM can help to make informed decisions with regard to funding socio‐behavioral research targeting AGYW for HIV prevention and research. This can further inform the development of research agendas and priorities.

#### 2.3.1 Existing EGMs and/or relevant systematic reviews

To the best of our knowledge, there are two existing EGMs (Portela et al., 2017; Rankin et al., 2016) (listed in Table 1) that partly focus on sexual and reproductive health issues of adolescents. In addition to this, we utilized Breakthrough ACTIONs SBC for HIV Evidence Database (Breakthrough ACTION, n.d.), which has consolidated evidence about the impact of SBC on HIV‐related outcomes—including HIV testing and counseling (HTC), voluntary medical male circumcision (VMMC), prevention of mother‐to‐child transmission (PMTCT), the treatment continuum, condom use, and other prevention areas—from 1999 to 2016.

**Table 1:** List of existing EGMs and/or relevant systematic reviews

Study/project name	Org| Date	Focus population	Geography	Scope of analysis
SBCE Interventions for Reproductive, Maternal, Newborn, Child Health (RMNCH)	3ie|2017	Women, children and adolescents	Global	Existing empirical evidence on the effects of key SBCE interventions to strengthen the individual, family, and community capabilities for RMNCH
Adolescent Sexual and Reproductive Health Evidence Gap Map	3ie|2016	Adolescents (boys and girls aged 10–19)	LMIC	Existing empirical evidence and gaps in evidence on the effects of SRH programming on adolescents in LMICs.

Abbreviations: EGM, evidence and gap map; LMIC, low‐ and middle‐income country.

However, there is no existing EGM which specifically looks at the use of diverse SBCC strategies among AGYW, aged 15–24, in LMICs for strengthening HIV prevention measures and facilitating participation in research.

## 3 OBJECTIVES

### 3.1 Objectives

This EGM aims to identify, map and describe existing evidence on SBCC strategies targeted at AGYW (15–24 years) in low‐ and middle‐income countries (LMICs) and assess the gaps in the evaluation of these strategies for HIV prevention and research. More specifically, this map seeks to:

1. Identify and map existing evidence and gaps in the use of diverse SBCC strategies to strengthen the adoption of HIV prevention measures and facilitate participation in research among AGYW in LMICs.
2. Identify areas where more interventions and evidence are needed to better inform the design of future SBCC strategies and programs for AGYW engagement in HIV prevention and research.

The map has been produced as per Campbell Collaborations “Guidance for the Production of Evidence and Gap Maps” (White et al., 2020). Evidence search was initiated in January 2021, and analysis of the map began in March 2021. Further additional studies were added in April 2021.

### 3.2 Snapshot of the EGM

**Figure 1:**
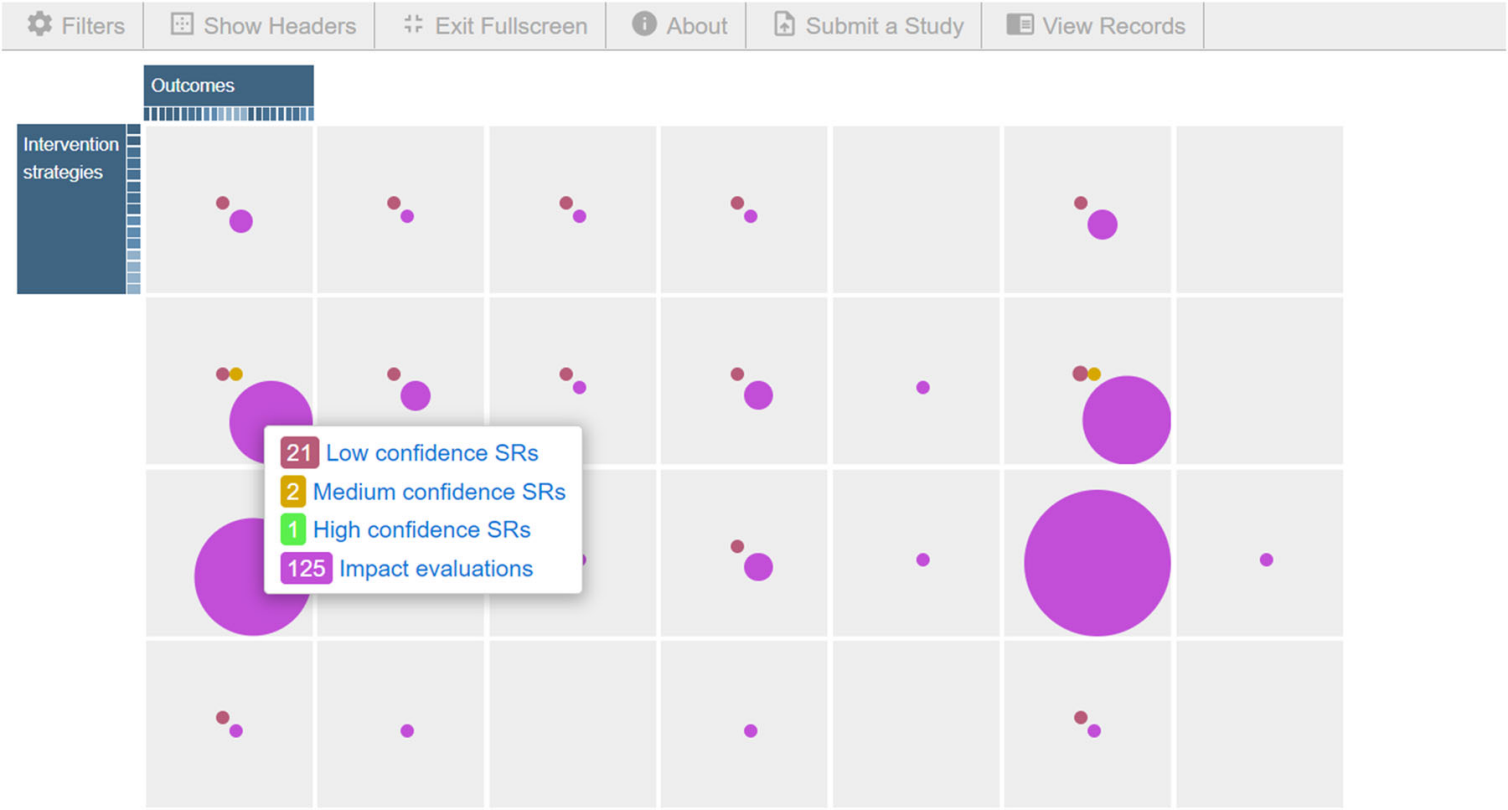
Snapshot of the evidence and gap map

The primary dimensions of the map include intervention and the outcome. The online map also shows the secondary dimension (filters) such as country, region, population, study confidence, and so forth (Figure 1). The pink bubbles indicate the number of impact evaluations assessing specific intervention and outcome strategies. The red, green, and yellow bubbles represent systematic reviews (SRs) with varying study confidence levels; red bubbles indicate low‐confidence SRs, green bubbles indicate high‐confidence SRs, and yellow bubbles indicate medium confidence SRs. The size of the bubbles indicates the volume of the evidence in that cell.

## 4 METHODS

### 4.1 EGMs: Definition and purpose

#### 4.1.1 What is an EGM?

An EGM is a decision‐making and research prioritization tool that highlights research gaps to inform strategic health and social policy, program, and research priorities (Snilstveit et al., 2013). Simply put, it provides a visual display of what we know and do not know by highlighting evidence that is either strong, weak, or non‐existent. EGMs are useful in avoiding needless duplication, and can also be used to identify where sufficient, high‐quality evidence from systematic reviews and randomized trials are available as a basis for decisions or where sufficient studies are available for knowledge synthesis (Snilstveit et al., 2016).

This EGM includes evidence from impact evaluations and systematic reviews. It is important to note that any single study may appear in multiple cells if it covers more than one category or sub‐category for either intervention or outcome.

##### Target population

This map focuses on “Adolescent Girls and Young Women” between the ages of 15–24, as defined by UNAIDS, in “low‐ and middle‐income countries, and their influencers.”

**LMICs:** LMICs are defined by the World Bank as low‐income economies—those with a Gross National Income (GNI) of $1035 or less in 2019; lower‐middle‐income economies—those with a GNI per capita between $1036 and $4045; and upper‐middle‐income economies – those with a GNI per capita between $4046 and $12,535 (2020).

##### Sub‐population: Urban and rural AGYW in formal and informal settlements

According to the United Nations Millennium Development Goals Report (2012), formal settings are characterized by adequate spatial planning and utilities (water, electricity, telephone, road, and other infrastructure). On the other hand, informal settings are defined as those with no urban development (e.g., slums), while in rural areas, these settings can encompass tribal areas. Informal settings can also include refugee camps in conflict or disaster‐affected areas in both rural and urban areas.

For this study, we looked at four kinds of settings:

- Urban formal—Formal housing settlements, formal institutions like schools/colleges, hospitals, religious places, offices and other areas under the municipality, etc.
- Urban informal—Slums/unauthorized settlements.
- Rural formal—Formal settlements and institutions like local governance systems, health centers, hospitals, religious institutions, commercial farms, community‐based organizations (CBOs), schools, etc.
- Rural informal—Community spaces like market areas, public meeting grounds, tea stalls, community fairgrounds, etc.

In addition to these, this EGM focuses on women living in a high‐risk environment/s, for example, sex workers, etc. Further, the map also includes the influence networks of AGYW, primarily male partners, family elders, peers, key community members, and service providers.

##### Types of interventions

The SBCC interventions included in this EGM are divided into four key categories ‐ mass media‐based interventions, community media‐based interventions, interpersonal communication, and Information and Communication Technology (ICT) and digital media‐based interventions. These interventions are further sub‐categorized, the details of which are given in the table below (Table 2).

**Table 2:** Intervention categories

Intervention	Intervention definition
Mass Media Interventions *Interventions that are characterized by expansive reach and are intended to reach a mass audience fall under mass media. These are large‐scale and usually quite cost‐intensive*.	
Print Media	Interventions that use print‐based material, such as books, newspapers, journals, comics, novels, posters, and brochures to disseminate textual and visual information to diverse audiences on a large scale.
Electronic Media	Interventions that use audio‐visual material, such as TV and radio to disseminate textual and visual information to diverse audiences on a large scale.
Community‐Based Interventions *Interventions that are designed for/with the community where the key population of interest resides. In this, interventions are more localized and contextualized and aim at achieving a community buy‐in*.	
Community Media	Community media‐driven interventions—such as community radio, video, newspapers, newsletters, and community screenings—encourage the participation of individuals, groups, or organizations through locally established and geographically specific media forms.
Folk Media	Interventions that use localized, traditional media in the form of music, drama, dance, and puppetry.
Theatre and arts‐based approaches	Interventions that use contextualized dramatic art forms to prompt community participation on specific issues.
Community Dialogues	Interventions that initiate open discussions and dialogues among participants and local groups through facilitated sessions that support self‐reflection around issues about HIV/SRH.
Capacity Strengthening	Interventions targeted at strengthening the knowledge, skills, and capabilities of both providers and recipients of health services to adopt/deliver health interventions effectively.
Gamification and experiential learning	Interventions that support people‐centric, hands‐on learning, through pedagogies such as game components, participatory learning include the use of score, challenge, and achievement to motivate and engage participants (Kolb, 1984).
Interpersonal Communication *Interventions that involve one‐to‐one or small group interaction and exchange*.	
Counseling	Interventions that use one‐on‐one communication with a trusted communicator or community leader such as a counselor, teacher, or health provider.
Home Visits	Interventions where the homes of target populations and their influencers are visited by peers and community health workers to engage with them in a one‐to‐one and confidential manner.
Peer‐led intervention	Interventions that use peer networks to ensure communication on a one‐to‐one basis, or in small and large groups
ICT and Digital Media‐Based Interventions *Interventions are characterized by the use of technology and digital content creation which can be disseminated over the internet or mobile networks*.	
Social media	Interventions that use social media platforms such as Facebook, YouTube, TikTok, and Instagram to disseminate information and encourage users to interact with each other and engage in dialogue on a large scale.
Mobile‐based services	Interventions that use mobile‐based services such as SMS and IVRS to disseminate information and encourage users to interact with each other and engage in dialogue on a large scale.
Digital games and learning tools	Interventions that use highly interactive and culturally relevant games and other tools through elements of roleplay and simulation to motivate users through sustained exposure.
Interactive app‐based services	Interventions that use interactive app‐based services, at scale, to disseminate information about HIV prevention and treatment and provide other resources for support.

In case the interventions studied did not exactly match the above‐mentioned categories, the team discussed and categorized them to their closest match based on the intervention definition and made a note of the same. Additionally, if a single study covered multiple interventions, the eligible interventions were coded and included.

##### Types of outcomes

**Figure 2:**
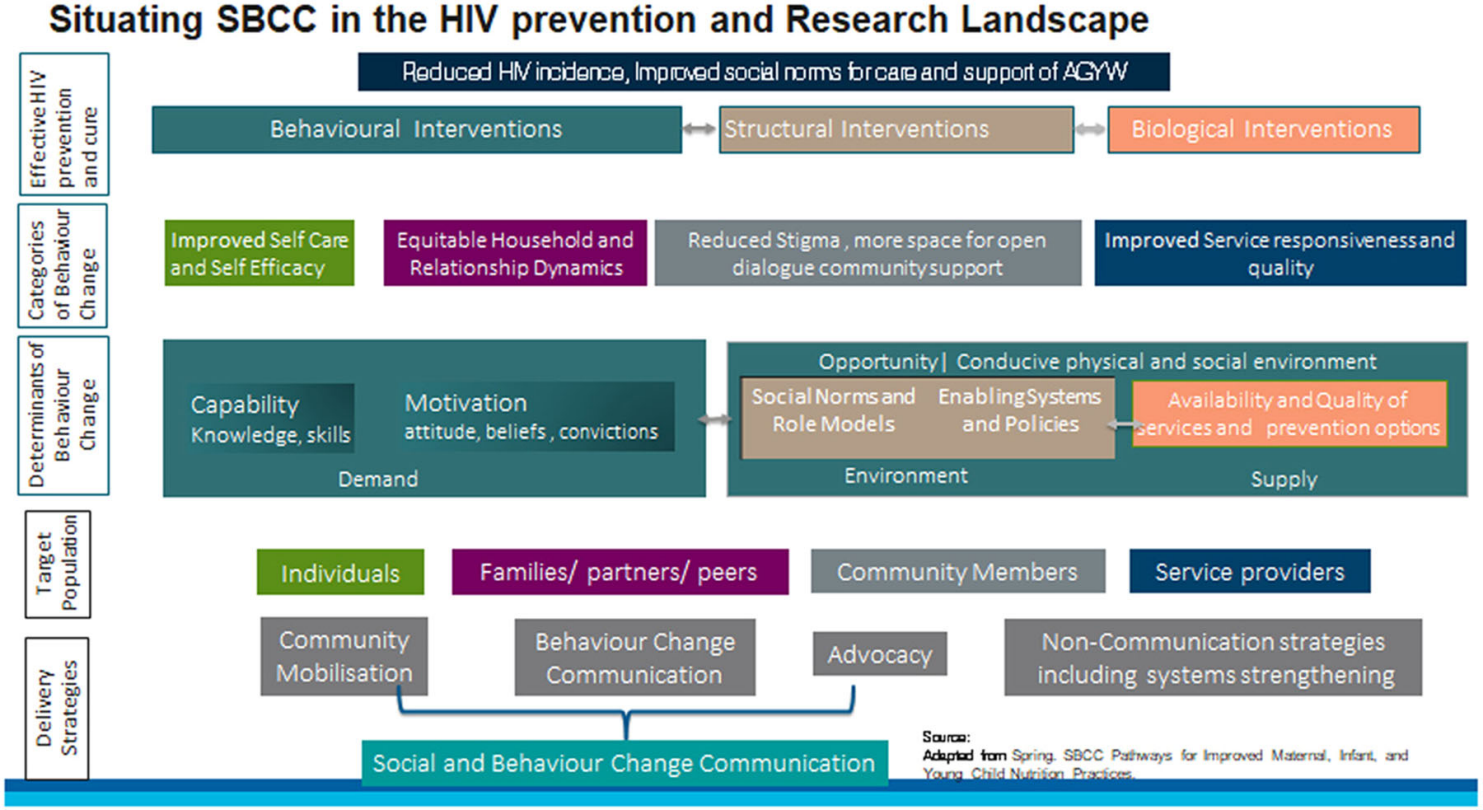
Conceptual framework

The outcome categories in this EGM include behavioral and health‐related outcomes, outlined along the causal chain based on the conceptual framework presented below (Figure 2). Behavioral outcomes were categorized at three levels—Individual (AGYW), Influencer (partner, household, and community), and Institutional (including actors like healthcare providers). Health outcomes were further divided into prevention‐related outcomes and research‐related outcomes. These outcomes were sub‐categorized based on the COM‐B model, Theoretical Domains Framework (TDF), and the Reproductive Empowerment Framework (2017). Table 3 summarizes the outcomes in detail.

**Table 3:** Outcome categories

Behavioral outcomes	Outcome definition
INDIVIDUAL	
Knowledge, Attitudes, and Skills	
1. Knowledge and awareness about HIV and STI	Level of understanding around HIV transmission, diagnostics, prevention, treatment, interventions (where to seek care and how to seek care, access points, etc.)
2. HIV/STI risk perception	Beliefs that individuals have about the characteristics and risks of HIV/STIs, and intention to adopt healthy behavior.
3. Trust in Healthcare providers/services	Level of confidence regarding available interventions and healthcare services, as well as the patient‐provider relationship (including counselors and peer educators).
4. Individual Agency and Self Efficacy	Ability to define desires, develop plans and take decisions to execute them.
5. Negotiation and life skills	Ability to exercise voice, and choice in interactions with peers, partners, family, and actors outside of immediate networks like healthcare providers, community leaders, etc.
INFLUENCERS	
Partner and Relationship Dynamics	
1. Partner's HIV/STI awareness	Measures addressing partner's knowledge of transmission, diagnostics, prevention, treatment, interventions (where to seek care and how to seek care, etc.)
2. Power equity and role in decision making	Measures addressing unequal gender and power relations
	E.g., Freedom of access or choice in using contraceptives, saying no to sex, etc.
3. Sexual and intimate partner violence	Measures addressing physical, verbal, and emotional abuse and harassment.
Household Dynamics	
1. Parent/in‐law communication	Measures related to the ability of AGYW to have open conversations among family members.
2. Joint decision making in households	This domain has been explored through the following indicators:
	Measures specifically related to parental figures only, e.g., domestic violence, knowledge about sexual relations, intimate partner violence, use of prevention options, access to care, family planning, etc. related outcomes.
	Measures related to the capacity of AGYW to be active contributors to decision‐making processes within the household.
	Measures related to the capacity of parental figures towards creating an enabling environment for AGYW to be active contributors to decision‐making processes within the household.
Social and Community Norms	
*Social processes that help individuals change their thoughts, feelings, or behaviors*	
1. Gender norms and expectations	Measures related to the understanding and deconstruction of gender roles in the context of sexual and reproductive health, especially with regard to expectations in the form of social pressure, group norms, social support, conformity, etc.
2. HIV/STI myths and misperceptions	Measures that help address or reduce HIV/STI‐related myths and misperceptions.
3. Stigma and discrimination	Measures that address negative opinions and behaviors towards AGYW living with, or affected by, HIV. Also include measures addressing self‐stigma.
4. Community support systems ‐peers, community leaders, community elders, etc.	Measures that help create an enabling ecosystem within communities through support networks. These may include CBOs, FBOs, peers, community elders, youth groups, etc.
INSTITUTIONAL	
Health Care Services	
*Outcomes addressing AGYW health‐related problems through a mix of public, private, community, and voluntary health provider services*.	
1. Provider sensitization and engagement skills	Measures that focus on enhancing provider knowledge and skills to make them sensitive and responsive to the healthcare needs of AGYW.
2. Quality of care and satisfaction with services	Measures that help deliver desired health outcomes for AGYW and build user‐friendly services. It is also related to the continuum of care in healthcare services
HEALTH OUTCOMES	
Prevention	
1. Limiting sexual partners	Measures that promote risk reduction strategies convey that having multiple partners is risky.
2. Correct and consistent condom use	Measures that address the correct and consistent use of condoms every time individuals engage in sexual activity with their partners.
3. Routine testing and status awareness	Measures that relate to regular HIV testing, especially for those at high risk, for them to be made aware of their HIV status and subsequently get treatment.
4. Uptake of PrEP/other Biomedical prevention options	Measures that address the uptake of PrEP (pre‐exposure prophylaxis), PEP (post‐exposure prophylaxis), and long‐acting antiretroviral (ARV) drugs and other available or upcoming prevention options.
5. Raised age of sexual debut	Measures that promote delaying sexual debut, especially for unmarried adolescent girls and women.
Research Engagement	
1. Research Awareness and Benefit Perception	This domain has been explored through the following indicators:
	Measures that address AGYW knowledge/awareness of HIV science, vaccines, and research pipeline for new prevention options, trials, and biomedical research for HIV‐related biomedical products.
	Measures that address influencers' knowledge/awareness of HIV science, vaccines, and research pipeline for new prevention options, trials of biomedical research for HIV‐related biomedical products.
2. Participation in Biomedical Research	This domain has been explored through the following indicators:
	Measures in perception change on benefits of biomedical research/clinical trials.
	Measures of AGYW's attitudes (negative/positive), beliefs, and perception towards enrollment/participation in biomedical research.
	Measures of influencers' attitudes (negative/positive), beliefs, and perceptions towards enrollment/participation in biomedical research.

### 4.2 Criteria for including and excluding studies

Table 4 provides an overview of the inclusion and exclusion criteria that was used to code evidence during the development of this EGM.

**Table 4:** Inclusion and exclusion criteria

Population	AGYW (15–24 years) and Influencers like parents, partners, in‐laws, healthcare providers	Sub‐Population
		Rural and Urban
		Formal and Informal
Geography	LMICs	
Topics of Interest	HIV/AIDS and other STIs Testing and uptake of prevention options including condoms, PrEP. Consistent uptake of ART Participation in Biomedical Research Intimate partner violence, sexual violence or gender‐based violence affecting HIV/SRH related health outcomes Behavioral factors such as relationship goals, sexual negotiations, gaps in knowledge, self‐efficacy, risk perception Stigma and discrimination related barriers Collective action, community support, community‐driven action Social and Gender Norms affecting SRH and HIV related behaviors	
Topics not of interest	Other structural factors affecting HIV acquisition in the AGYW population ‐ School attendance, labor migration, child sexual abuse, lack of economic empowerment, structural barriers to accessing sexual and reproductive health services, marriage patterns, etc.	
	Biological factors	
	Other adolescent health issues like mental health, smoking, alcoholism, drug addiction, nutrition, etc.	
	Maternal health and childcare	
	Pregnancy, Family planning (if they do not measure effects of HIV/SRH outcomes).	
Study Type	Impact Evaluation (for which there was a control group)‐, Randomized Controlled Trials (RCT), Controlled Before and After, Cross‐Sectional or Panel studies, Mixed Method studies that combine any of the above designs with qualitative research	
Time Frame	Studies published from 2000 onwards	
Language	English	

### 4.3 Search sources

The studies for this EGM were searched using a comprehensive search strategy (detailed in Supporting Information: Appendix A). A pilot search strategy was developed based on the selection of studies that met the inclusion criteria (Table 4). This was done under the guidance of an information specialist associated with the Campbell Collaboration. The search was conducted using various databases which include SCOPUS, PubMed, PsychINFO, EBSCOhost, Popline. In addition to databases, institutional repositories and websites such as International AIDS Society (IAS), HIV Research for Prevention (HIVR4P), AIDS and SBCC conference, Johns Hopkins Center for Communication Programs (JHU CCP), Tata Institute of Social Sciences (TISS), Harvard, The Elizabeth Glaser Paediatric AIDS Foundation (EGPAF), FHI 360, Palladium, Kimonics, Delloite, University Research Co. (URC), Population Services International (PSI), International Center for Research on Women (ICRW), BBC Media Action, Karnataka Health Promotion Trust (KHPT), HIV AIDS Alliance, NACO‐National Integrated Biological and Behavioural Surveillance (NACO‐IBBS), BCI, Health Communication Capacity Collaborative (HC3), and Compass were searched. Existing evidence and gap maps, such as the International Initiative for Impact Evaluations (3ie) Adolescent Sexual and Reproductive Health Evidence and Gap Map, were also searched. Further, reference lists of existing systematic reviews and primary studies were searched for relevant studies. Abstracts of the AIDS conference from 2000 to 2020 were accessed from year‐wise abstract books, as well as the online resource library of the International AIDS Society. Abstracts of the papers presented at HIVR4P from 2014 onwards were also screened to identify any eligible studies. The included studies in the map were identified from January 2021 to April 2021

### 4.4 Types of evidence

We included studies that assess the effects of interventions using experimental designs or quasi‐experimental designs with non‐random assignments that allow for causal inference (see criteria below). Systematic reviews on the effect of interventions were also included, along with cross‐sectional and panel studies^1^ in addition to the designs that allow for causal reference.

As defined by Moher et al. (2015), “A systematic review attempts to collate all relevant evidence that fits pre‐specified eligibility criteria to answer a specific research question. It uses explicit, systematic methods to minimize bias in the identification, selection, synthesis, and summary of studies. The key characteristics of a systematic review are (a) a clearly stated set of objectives with an explicit, reproducible methodology; (b) a systematic search that attempts to identify all studies that would meet the eligibility criteria; (c) an assessment of the validity of the findings of the included studies (e.g., assessment of risk of bias and confidence in cumulative estimates); and (d) systematic presentation, and synthesis, of the characteristics and findings of the included studies”.

The key characteristics for a review to be included as a “systematic review”

1. A clearly stated set of objectives with pre‐defined eligibility criteria for studies.

1. An explicit, reproducible search strategy.
2. A systematic search that attempts to identify studies that would meet the eligibility criteria.
3. A systematic presentation, and synthesis, of the characteristics and findings of the included studies.

We included impact evaluations that met the following criteria:

Experimental and quasi‐experimental studies:

- Studies where participants are randomly assigned to treatment and comparison groups (experimental study designs).
- Studies where assignment to treatment and comparison groups is based on other known allocation rules, including a threshold on a continuous variable (regression discontinuity designs) or exogenous geographical variation in the treatment allocation (natural experiments).
- Studies with non‐random assignment to treatment and comparison groups that include pre‐and post‐test measures of the outcome variables of interest to ensure equity between groups on the baseline measure, and that use appropriate methods to control for selection bias and confounding. Such methods include statistical matching (e.g., propensity score matching, or covariate matching), regression adjustment (e.g., difference‐in‐differences, fixed effects regression, single difference regression analysis, instrumental variables, and “Heckman” selection models).
- Studies with non‐random assignment to treatment and comparison groups that include post‐test measures of the outcome variables of interest only and use appropriate methods to control for selection bias and confounding, as detailed above. This includes pipeline and cohort studies.

Panel studies: Ferraro and Miranda (2014, 2017) argue that combining panel data with baseline observations and statistical matching is the most effective quasi‐experimental method of reducing bias when evaluating conservation sector programs. However, given the expected small size of the evidence base, we included studies with post‐intervention outcome data only if they use some method to control for selection bias and confounding.

Cross sectional studies: Studies where paricipants are selected on the basis of the inclusion and exclusion criteria, after which the investigator follows the study and measures outcomes and exposures simultaneously (Setia, 2016)

Excluded study designs: Before‐after studies and observational studies without control for selection bias and confounding were excluded. Additionally, modeling‐based studies, commentaries, and literature reviews were excluded.

### 4.5 EGM protocol

The EGM protocol was published on January 18, 2022 (Bose et al., 2022).

### 4.6 Stakeholder engagement

An advisory group comprising nationally and internationally recognized domain experts—including bioethicists, researchers, scientists, social scientists, experts in health education, gender mainstreaming, Good Participatory Practices (GPP), as well as advocacy and communication professionals—was convened.

Experts were consulted at four key stages during the development of this EGM:

1. At the inception to assist in defining the scope and review the draft conceptual framework.
2. During the review of search methods and strategies.
3. During the review of preliminary results where they also provided additional information on how to locate further relevant studies.
4. At the time of review of the draft report.

A detailed list of the advisors and experts has been included in Supporting Information: Appendix C.

### 4.7 Dimensions

#### 4.7.1 Scope of the EGM

This EGM focuses on mapping available evidence about various kinds of SBCC interventions used for engaging AGYW in HIV prevention and research. Further, it helps understand behavioral determinants targeted by these interventions among AGYW towards desired social and health outcomes. The other actors who influence these behavioral determinants (referred to as influencers in this report) were also explored through the EGM.

Based on the framework mentioned below, the substantive scope of the EGM was delineated along the following key categories:

- Interventions consisting of SBCC intervention strategies.
- Outcomes consisting of behavioral, health, and research outcomes. Behavior change dimensions, as mentioned above, were guided by the COM‐B model and The Theoretical Domains Framework (TDF). The health and research outcomes were defined based on programmatic and research priorities, as designated by UNAIDS (2020a; 2020b), The Global Fund (2018), and the World Health Organization (WHO) (2020).

To keep the scope manageable, the EGM focused on the following topics of interest around HIV/AIDS and other STIs:

- Testing and uptake of prevention options including condoms, PrEP (pre‐exposure prophylaxis), PEP (post‐exposure prophylaxis), and long‐acting antiretroviral (ARV) drugs and other available or upcoming prevention options.
- Consistent uptake of Antiretroviral Therapy (ART).
- Participation in biomedical research.
- Intimate partner violence, sexual violence, or gender‐based violence affecting HIV and SRH‐related health outcomes.
- Behavioral factors affecting access to sexual and reproductive health services, such as relationship goals, sexual negotiations, gaps in knowledge, self‐efficacy, and risk perception.
- Stigma and discrimination related barriers.
- Collective action, community support, community‐driven action, and social and gender norms affecting SRH and HIV‐related behaviors.

This EGM does not focus on other structural factors affecting HIV acquisition in the AGYW population, such as school attendance, labor migration, child sexual abuse, lack of economic empowerment, structural barriers to accessing sexual and reproductive health services, marriage patterns, etc. Other factors impacting adolescent health such as mental health, smoking, alcoholism, drug addiction, nutrition, maternal health, and childcare are also outside of the purview of this EGM. Additionally, topics related to pregnancy, family planning, and abortions were not included in this study if they did not measure the effects of HIV and SRH‐related outcomes.

The interventions and outcomes along with their sub‐categories have been outlined in detail in Section 4.5.

#### 4.7.2 Conceptual framework

SBCC uses behavior‐centered communication approaches to help inform, persuade and provide support to individuals, households, peers, and communities towards creating an enabling environment across the continuum of HIV care, starting with prevention (Van Lith et al., n.d.). SBCC strategies are informed by multiple disciplines including the social sciences, behavioral sciences, behavioral economics, human‐centered design, and other theories of behavior change that address various determinants of behavior and the environment within which change occurs. SBCC interventions can be further categorized under three broad strategies: social and community mobilization, behavior change communication, and advocacy (Lamstein et al., 2014).

The conceptual framework (Figure 2) illustrates how SBCC strategies can contribute toward navigating behavioral pathways within complex systems to ensure global objectives of reduced HIV incidence and equip AGYW to negotiate social norms for the betterment of their health outcomes.

To achieve the above, there is a need for a mix of behavioral, structural, and biological interventions. The success of these interventions is dependent on multiple intrinsic and extrinsic behavioral factors constantly interacting with each other. Towards delineating some of these factors, we used the COM‐B model (Capability, Opportunity, Motivation, and Behavior model) proposed by Michie et al. (2014) and the Theoretical Domains Framework (TDF), which offers a comprehensive framework to identify factors that may directly influence behaviors, as well as desired behavior changes (Khan & Roche, 2018). This was further categorized based on the work of Marsh et al. (2008) who suggested that the adoption of behavior is dependent on three basic determinants—demand, environment, and supply.

Given the complex systems within which behavior change happens, we identified four key target groups that can have a direct or indirect influence on an individual's agency and decision‐making power for adopting behavior changes or their willingness for change. These include AGYW as individuals, their immediate relations (e.g., family, partner, peers), actors who might be outside the immediate circle of influence and yet significantly influence decision making (e.g., community members like religious and key opinion leaders and service providers from healthcare systems).

Lastly, the framework focuses on SBCC delivery strategies, which are a mix of community mobilization, behavior change communication, and advocacy strategies. While other non‐communication strategies such as delivery and access to services are also critical for behavior change, the focus of this EGM remains on SBCC‐based delivery strategies.

### 4.8 Description of intervention

Table 2 lists the intervention categories and sub‐categories.

### 4.9 Type of population

This study focuses on “Adolescent Girls and Young Women” as defined by UNAIDS (2019), between the ages of 15–24, in low‐ and middle‐income countries, and their influencers. Population sub‐categories include pregnant women and new mothers, sex workers, and people living with HIV (PLHIV).

### 4.10 Types of location

As mentioned above, this EGM focuses on AGYW populations in low‐income and lower‐ and middle‐income countries, as defined by the World Bank

### 4.11 Types of settings

This map looks at four kinds of settings—urban formal, urban informal, rural formal, and rural informal. Informal settings may also include refugee camps in conflict or disaster‐affected areas in both urban and rural contexts.

## 5 DATA COLLECTION AND ANALYSIS

### 5.1 Data collection process

Two researchers independently coded the studies. Any discrepancies in the decision of the two researchers were resolved through discussion. If the disagreements persisted, a third researcher intervened. A piloted coding form with information necessary to generate the EGM, such as intervention strategies, outcome categories, and other filters, was used to code the studies. The full bibliographic information of the included studies is recorded in reference section.

#### 5.1.1 Screening and study selection

The screening of the studies was done at the title and abstract level, as well as at the full‐text level, based on intervention, study design, and population. Two researchers independently screened the studies at both stages (title and abstract screening stage). Outcomes were not included in this process. Subsequently, full texts of eligible studies were retrieved and screened, and the reviewers compared the results. The authors of the studies were not contacted at any point for missing information.

#### 5.1.2 Inclusion decisions

Two people independently screened the records at the title and abstract stage and the full‐text stage using a screening tool containing inclusion and exclusion criteria. Screening decisions were matched, and in case of any disagreements, an arbitrator resolved these issues at the level of screening.

#### 5.1.3 Data extraction and management

Two reviewers extracted data on both published and ongoing randomized trials related to population, interventions, comparisons, and outcomes. Coding categories (Supporting Information: Appendix E) are based on the intervention and outcome framework. Information on additional filters, such as country of study (using the World Bank country classifications by income), study design (e.g., RCT or systematic review), age group (15–24 years), and setting (urban and rural and formal and informal settings, as defined by the United Nations), are included.

#### 5.1.4 Tools for assessing the risk of bias/study quality of included reviews

The purpose of the EGM was to identify all available impact evaluations and systematic reviews. The quality of the systematic reviews/meta‐analyses was assessed with AMSTAR‐2 (Shea et al., 2017). The risk of bias was not assessed for primary studies (Snilstveit et al., 2017). To account for the differences in the quality of study designs and analysis methods, we appraised the risk of bias in all included systematic reviews by the risk of bias status. Studies with both negative and positive outcomes were considered for the above‐mentioned designs.

### 5.2 Analysis and presentation

#### 5.2.1 Report structure

This report contains tables and figures indicating the number of included studies, their geographical‐ and population‐wise distribution, along with intervention and outcome categories and sub‐categories. Period of publication, study type, and funding agency are some of the other filters covered in this report. Narrative descriptions of the same are provided as well.

#### 5.2.2 Presentation

The primary dimensions of the report include interventions and outcomes, as described earlier. Secondary dimensions or filters include region, country, year of publication, study setting, population sub‐groups, and funding agency.

#### 5.2.3 Dependency

The unit of analysis is each paper. However, in cases where there are multiple versions of the same paper, the latest or most complete version is used for the analysis.

## 6 RESULTS

### 6.1 Results of the search

For this EGM, a total of 2121 records were retrieved through a database search. Further, 381 additional records were identified through a gray literature search which included website searches, references of included studies, and conference abstract books from AIDS (2000–2020) and HIVR4P (2014 onwards).

Of the total 2502 records, 89 duplicates were further removed and a total of 2413 records were included for title and abstract screening.

**Figure 3:**
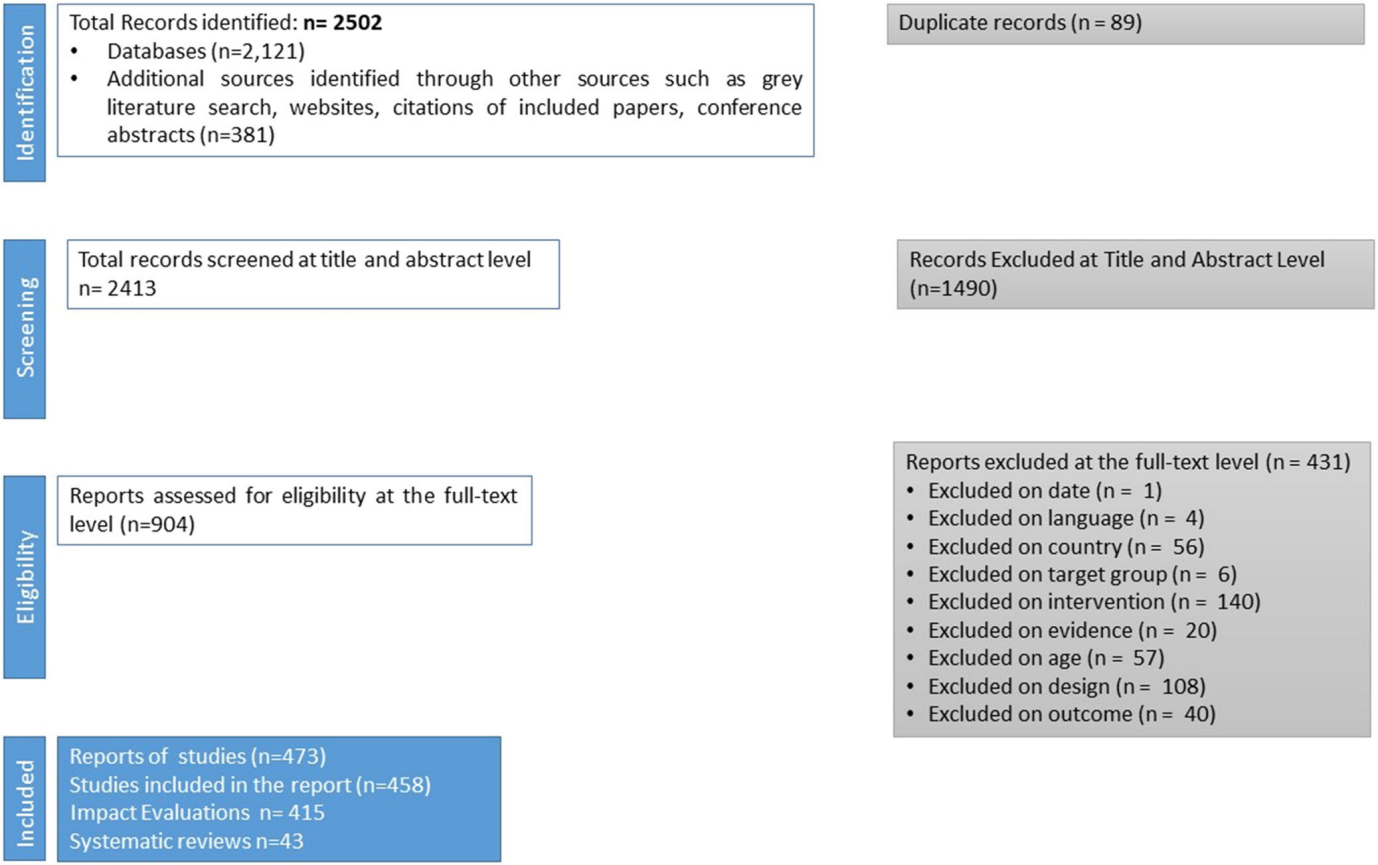
PRISMA diagram

Of these 2413 records, 904 reports were screened for full text. A total of 431 reports were excluded at the full‐text screening stage. Screening at the full‐text stage resulted in the inclusion of 473 records. The reasons for the exclusion of records are given in the PRISMA flowchart (Figure 3). Examples of the 431 excluded studies have been described with reasons in Section 15.2.

This report is based on the analysis of 458 coded studies—415 impact evaluations and 43 systematic reviews—as studies with multiple papers were linked and the most complete or latest version was used for the analysis.

### 6.2 Distribution of evidence: Primary dimensions

#### 6.2.1 Evidence base by intervention

**Figure 4:**
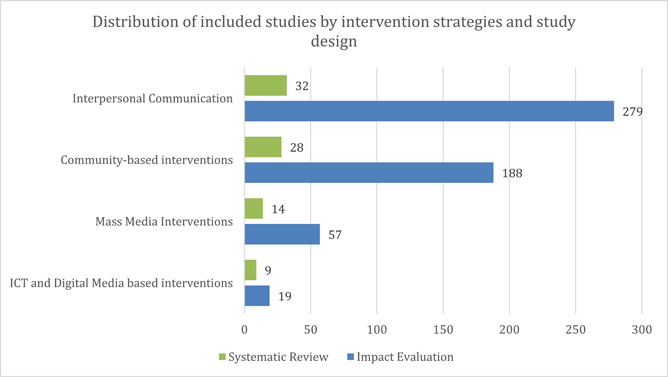
Evidence segmentation as per intervention categories and study design

The evidence base shows interpersonal communication as the most studied intervention category, with 311 studies, followed by community‐based interventions (*n* = 216) as seen in Figure 3. Mass‐media interventions were relatively less studied (*n* = 71) along with ICT and digital media‐based interventions (*n* = 28) (Figure 4).

#### 6.2.2 Evidence as per intervention sub‐categories

**Figure 5:**
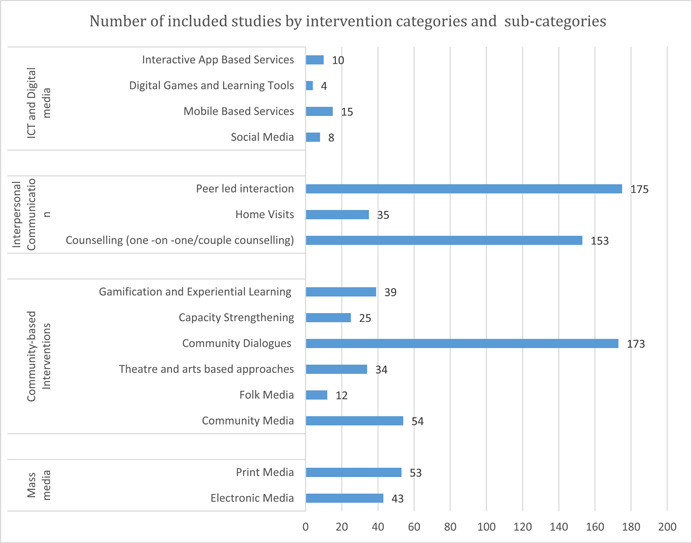
Included studies by intervention categories and sub‐categories

Figure 5 presents the distribution of evidence according to SBCC intervention sub‐categories. Within the interpersonal communication category, which is amongst the most evidenced, the sub‐category of peer‐led interactions (*n* = 175) was the most dominant intervention type, followed by counseling (*n* = 153). However, very few studies focused on home‐visit‐based intervention methods (*n* = 35). In the community‐based interventions category, most of the studies focused on community dialogues (*n* = 173), while emphasis on other sub‐categories—community media (*n* = 54), gamification and experiential learning (*n* = 39), theatre and arts‐based approaches (*n* = 34), capacity strengthening (*n* = 25), folk media (*n* = 12)—was comparatively lower

While the mass‐media intervention category was comparatively less evidenced than other intervention categories, its sub‐categories—electronic media (*n* = 53) and print media (*n* = 43)—were comparatively better studied than other intervention sub‐categories like folk media, gamification, and experiential learning, theatre, and arts‐based approaches, among others.

ICT and digital media‐based interventions were least evidenced among all intervention categories. Subsequently, most of its sub‐categories remain poorly studied, including mobile‐based services (*n* = 15), interactive app‐based services (*n* = 10), social media (*n* = 8), and digital games and learning tools (*n* = 4).

It is important to note that studies with multiple interventions have been coded across multiple sub‐categories and hence the figures presented in the graph below (Figure 5) might not correspond to the graph above (Figure 4).

#### 6.2.3 Evidence as per outcome category

**Figure 6:**
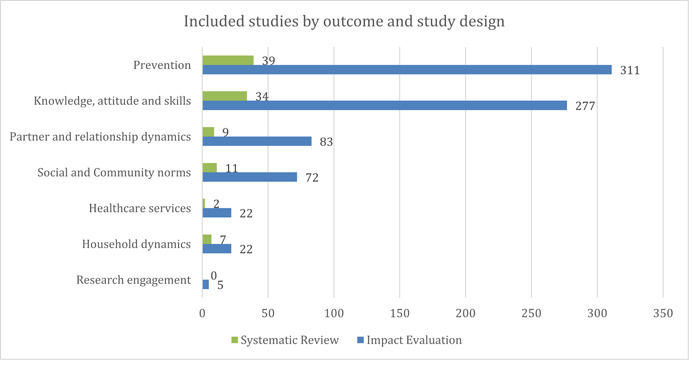
Included studies by outcomes and study design

As seen in Figure 6, prevention‐related outcomes (*n* = 350) had the highest number of studies among all outcome categories, followed by studies on knowledge, attitude, and skills (*n* = 311). Studies with a focus on strengthening partner/relationship dynamics (*n* = 92) and addressing social and community norms (*n* = 83) were relatively lower. The least evidenced outcome categories included household dynamics (*n* = 29), healthcare services (*n* = 24), and research engagement (*n* = 5).

#### 6.2.4 Evidence as per outcome sub‐categories

Given that SBCC interventions have been mostly targeted toward strengthening prevention‐related outcomes, it is not surprising to see that correct and consistent condom use (*n* = 296) remains a densely studied sub‐category, along with limiting sexual partners (*n* = 162), as seen in Figure 6. Further, given global efforts to amplify testing and treatment, we also see evidence for routine testing and status awareness (*n* = 92) being relatively better studied, while outcomes related to strengthening uptake of newer prevention approaches like PrEP and other biomedical prevention options (*n* = 13) have been less studied within this category.

Knowledge attitude and skills remain the second most evidence outcome category, with a bulk of evidence concentrated on interventions targeting outcomes related to knowledge and awareness about HIV/STIs (*n* = 280) and HIV/STI Risk Perception (*n* = 144). However, only 79 studies focused on strengthening individual agency and self‐efficacy. Further, while interventions focused on knowledge and attitude are better populated, those targeting outcomes like negotiation and life skills (*n* = 9) and trust in healthcare providers/services (*n* = 4) are sparsely populated.

Outcomes aimed at addressing partner and relationship dynamics remain comparatively less studied. A deep dive into its sub‐categories highlights the need for more substantiated evidence in this category—addressing sexual and intimate partner violence (*n* = 48), power equity and role in decision making (*n* = 34), and partner's HIV/STI awareness (*n* = 32).

Outcomes related to social and community norms were also relatively less studied—stigma and discrimination (*n* = 41), community support systems (*n* = 35), gender norms (*n* = 13), HIV/STIs myths, and misconceptions (*n* = 5). Outcomes targeted at healthcare services were also minimal—quality of care/satisfaction with services had 16 studies while provider sensitization and engagement skills had 12.

Household dynamics was one of the lesser evidenced outcome categories, with only 27 studies focusing on parent/in‐law communication and just two studies focusing on joint decision making in households.

**Figure 7:**
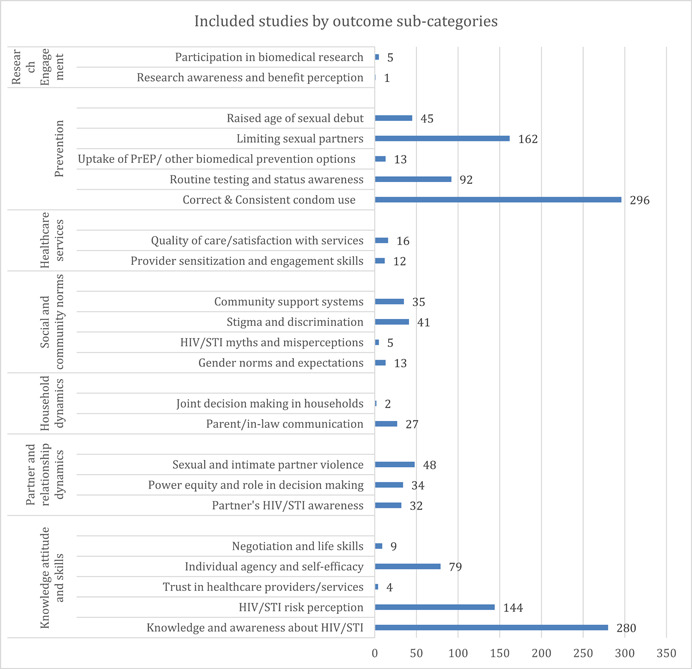
Included studies by outcome categories and sub‐categories

Finally, research engagement as an outcome category was least studied, with just five studies focusing on outcomes related to participation in biomedical research and just one study targeting research awareness and benefit perception (Figure 7).

### 6.3 Distribution of evidence: Secondary dimensions

#### 6.3.1 Distribution of evidence based on region

##### Regional distribution of evidence

**Figure 8:**
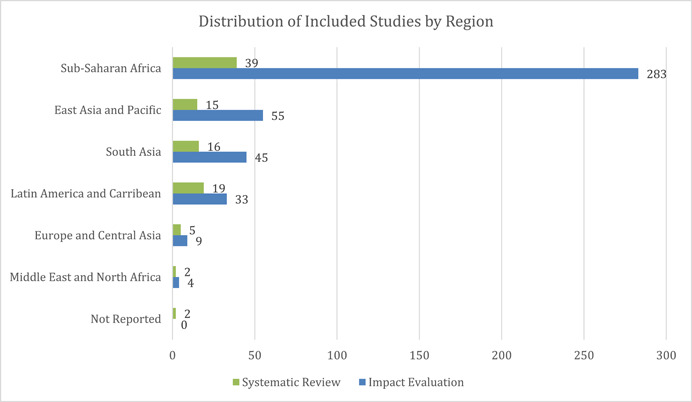
Distribution of included studies by region and broad study design. The number of studies does not equal the total number of studies as a single study may be coded as more than one study design (mostly a process evaluation with RCT/any other effectiveness design).

Sub‐Saharan Africa is the most well‐represented region, constituting about 70% (*n* = 322) of all the included studies, followed by East Asia and Pacific with 15% of studies (*n* = 70), and South Asia with 13% of studies (*n* = 61). Included studies from the Latin America and Caribbean region constituted about 11% (*n* = 52) of the total included studies, while the Middle East and North America (MENA) region had the lowest number of included studies (*n* = 6). The distribution of included studies across various regions and study designs, from 2000 till April 2021 has been represented in Figure 8.

*Please note: Since some studies had both an impact evaluation/different effectiveness designs and process evaluations, they appear in more than one place in the table*.

**Figure 9:**
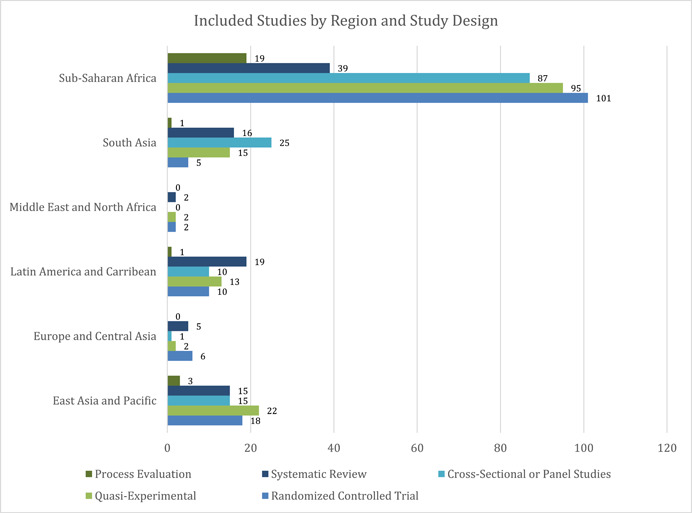
Distribution of included studies by region and study design sub‐categories

Additionally, distribution of evidence across study design (Figure 9) suggests that quasi‐experimental studies were the most used methods across regions (*n* = 149), followed by randomized controlled trials (RCTs) (*n* = 142) and cross‐sectional or panel study design (*n* = 138). However, trends vary across region—in Sub‐Saharan Africa, the most used study design is RCTs, in South Asia, most studies use cross‐sectional or panel study design, in East Asia and the Pacific a majority of studies use quasi‐experimental study designs, and, in Latin America systematic reviews are the predominant study design.

##### Country‐wise distribution of included studies by design

**Figure 10:**
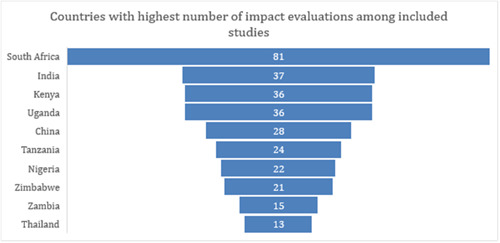
Top 10 countries with the highest number of impact evaluations

South Africa has the highest number of impact evaluations (*n* = 81) among all countries, as highlighted in Figure 10. Some of the other countries in the Sub‐Saharan Africa region with a high number of included impact evaluations are Kenya (*n* = 36), Uganda (*n* = 36), Tanzania (*n* = 24), Nigeria (*n* = 22), Zimbabwe (*n* = 21), and Zambia (15). In the East Asia and Pacific region, China and Thailand have 28 and 13 impact evaluations, respectively. India has the highest number of studies (*n* = 37) in the South Asia region.

**Figure 11:**
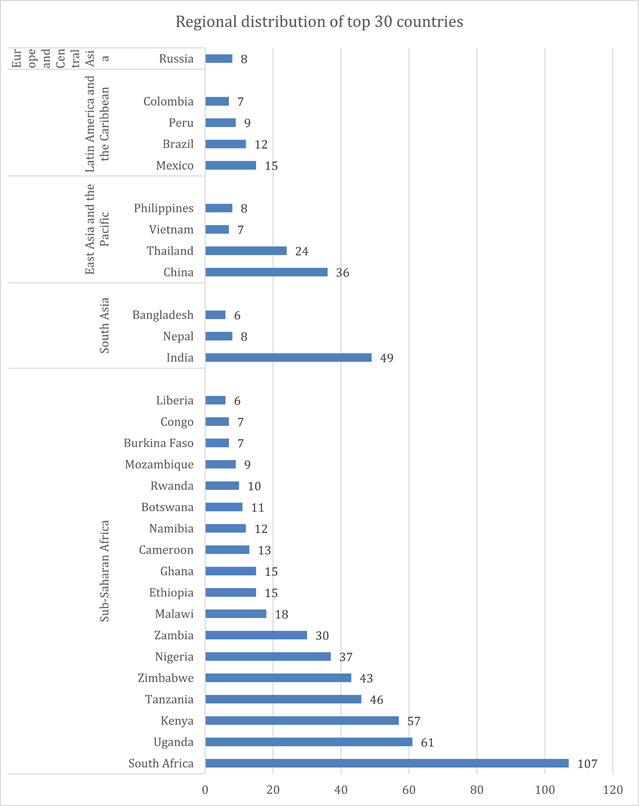
Top countries with the highest number of impact evaluations

Figure 11 presents a glimpse of the regional distribution of top 30 countries with the highest concentration of evidence, including both systematic reviews and impact evaluations.

**Figure 12:**
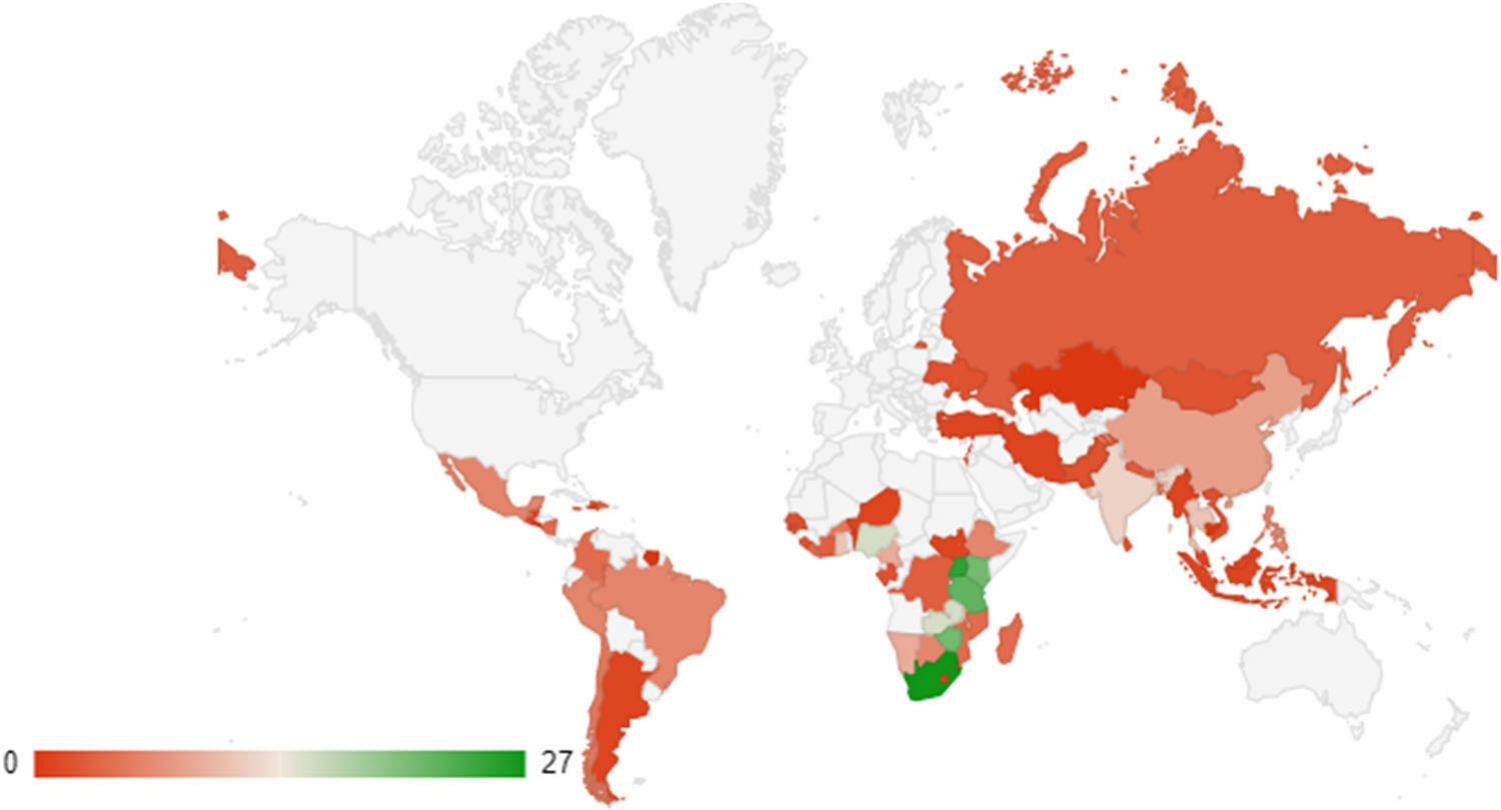
Heat map of systematic reviews

**Figure 13:**
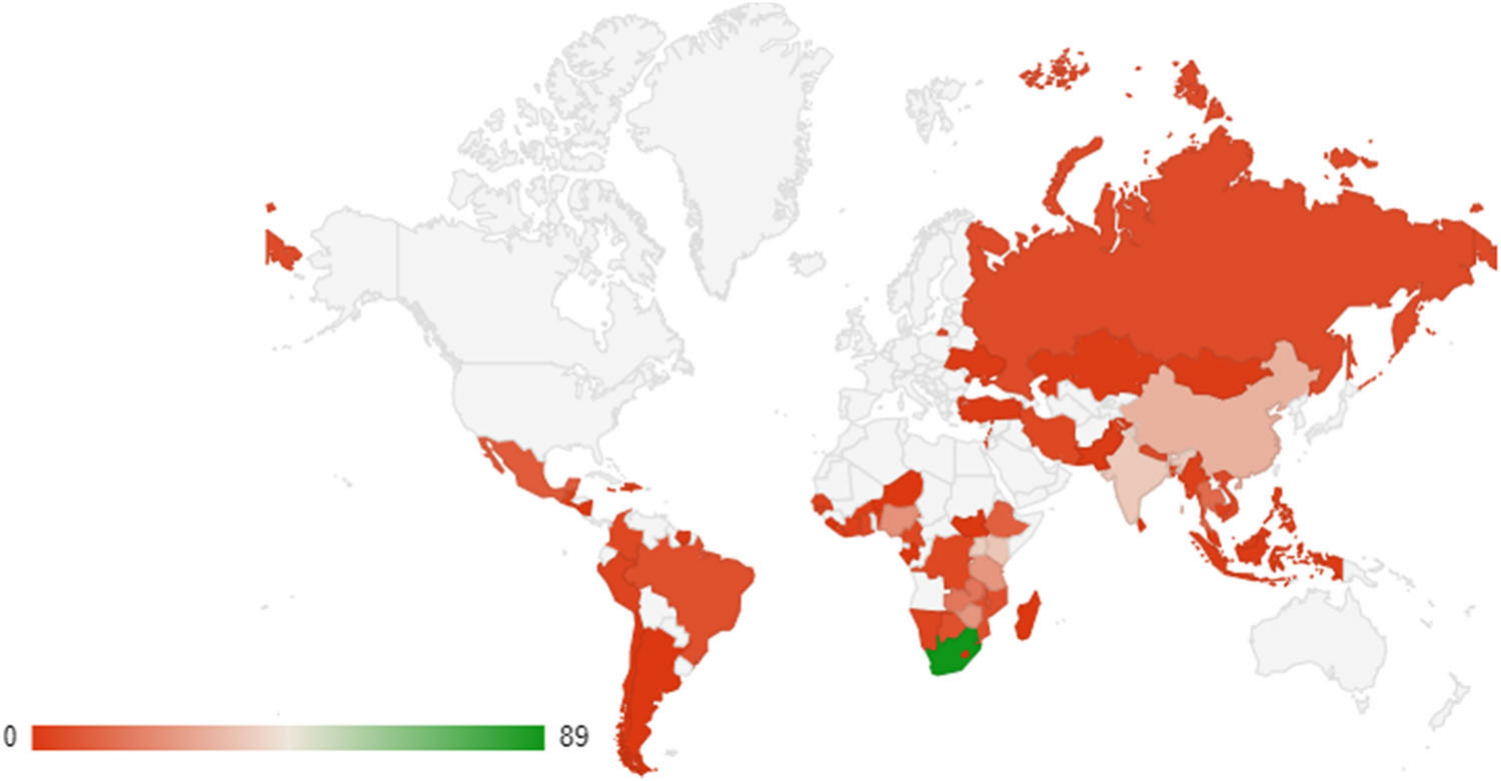
Heat map of impact evaluations

The country‐wise distribution of included studies for systematic reviews and impact evaluations are also shown as heat maps in Figures 12 and 13. In these figures, countries highlighted in green represent those with the highest volume of studies, and those in red contain a relatively lower volume of studies.

The full, country‐wise distribution of included studies and their respective study designs is available in Supporting Information: Appendix B.

Figure 11 presents a glimpse of the regional distribution of top 30 countries with highest concentration of evidence including both systematic reviews and evaluation.

##### Publication period and study design

**Figure 14:**
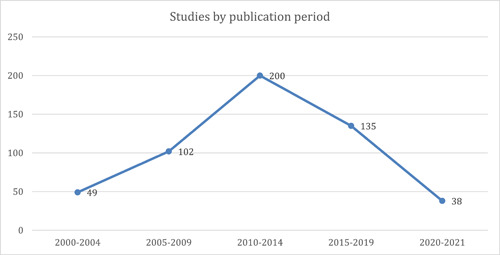
Studies by publication period

The evidence base for these studies seems to significantly increase from 2005 to 2014. The graph (Figure 14) hits its peak between 2010 and 2014 (*n* = 200), after which a slight dip in evidence generation is witnessed between 2015 and 2019. While the graph below shows a further decrease in the number of studies following the 2015–2019 period, this is a 2‐year interval which cannot be compared to the rest of the evidence timeline reporting a 5‐year period.

**Figure 15:**
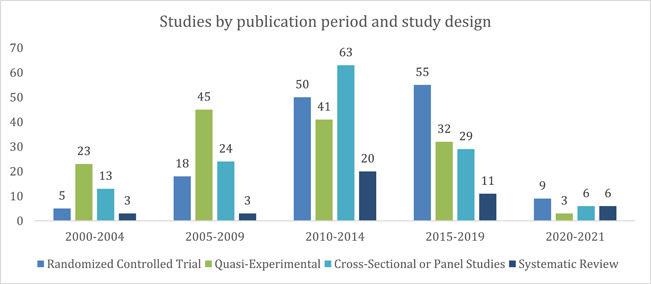
Included studies by publication period and study design

In terms of study design, we see a growing trend for RCTs; in 2000–2004, only five studies were using the RCT technique, however, between 2015 and 2019, the total number of studies using RCT grew to 55 (Figure 15). Additionally, while quasi‐experimental study design was most used (*n* = 45) in 2005–2009, over the years a lesser number of studies have adopted this design. The number of cross‐sectional or panel studies published was highest from 2010 to 2014 (*n* = 63), however, we see a sharp decline in the use of this design between 2015 and 2019 (*n* = 29). The highest number of systematic reviews (*n* = 20) was published between 2010 and 2014, a significant jump from 2000 to 2004 and 2005–2009 when an equal number of systematic reviews (*n* = 3) were published. However, between 2015 and 2019 only 11 systematic reviews were produced.

##### Publication period and region

**Figure 16:**
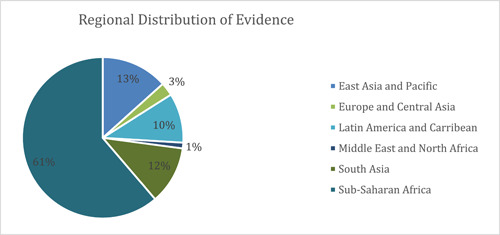
Included studies by region

More than 60% (*n* = 321) of all included studies in this EGM are from Sub‐Saharan Africa (Figure 16). The predominance of studies from this region is likely driven by the higher burden of HIV in the region. However, evidence across other regions remains sparsely populated. These include East Asia and the Pacific region (13%, *n* = 70), South Asia (12%, *n* = 61) followed by Latin America and the Caribbean (10%, *n* = 52). The least populated regions include LMICs in Europe and Central Asia (3%, *n* = 14) and the Middle East and North Africa region (1%, *n* = 6).

**Figure 17:**
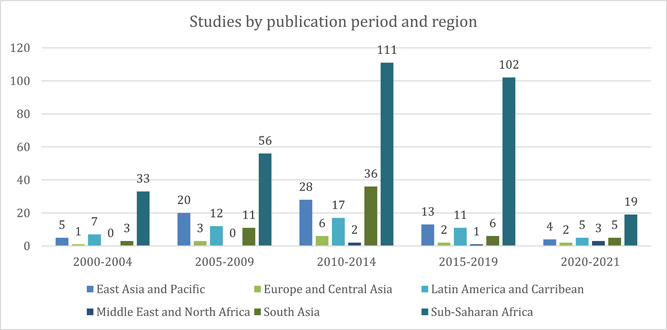
Included studies by region and publication period

A deeper dive into the evidence based on publication period and region (Figure 17) highlights that, across all regions, there has been a consistent increase in studies till 2014. However, between 2015 and 2019, there was a dip in the number of studies published. We also see that, while the East Asia and the Pacific region has been the second most studied, South Asia saw a significant jump between 2010 and 2014, with 36 studies published within this period, overtaking East Asia and the Pacific as the second‐highest evidenced region. However, from 2015 to 2019, only 6 studies were published from South Asia, while 13 were published from East Asia and the Pacific.

#### 6.3.2 Distribution of evidence across population and sub‐types

##### Distribution of evidence across populations

**Figure 18:**
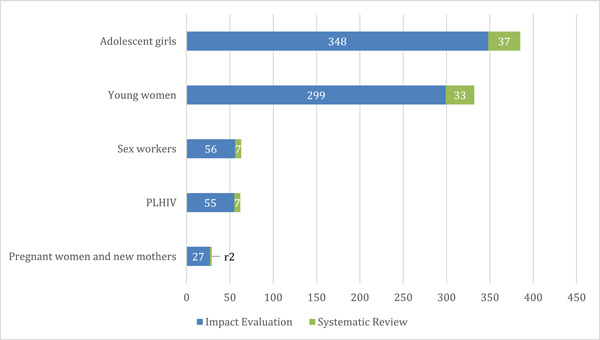
Included studies by population sub‐groups and study design

A majority of impact evaluations focused on interventions targeted at adolescent girls (*n* = 348) and young women (*n* = 299), as seen in Figure 18. Impact evaluations for sub‐groups like sex workers (*n* = 56) and people living with HIV (PLHIV) (*n* = 55) were low, while only 27 studies focused on pregnant women and new mothers (PWNM).

##### Distribution of evidence across settings

**Figure 19:**
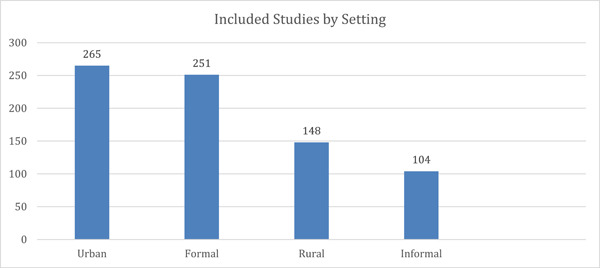
Included studies by setting

Evidence highlights that a majority of studies were conducted in informal (*n* = 251) and/or urban settings (*n* = 265), as seen in the graph below (Figure 19). Evidence from rural (*n* = 148) and informal (*n* = 104) settings was relatively lower. It may be noted here, however, that in some of the studies, interventions were conducted in more than one setting. For example, an intervention conducted in a school in a rural area was coded under the categories rural and formal (e.g., Harrison et al., 2016). Some of the studies were conducted among patients receiving services at clinics in a city (e.g., Abdala et al., 2013; Myerson et al., 2012) while others were conducted in schools, universities, or other educational institutes in a city (e.g., Baghianimoghadam et al., 2012; Boti et al., 2019). The setting in such studies was coded as urban and formal. Finally, some of the studies included both urban and rural settings (e.g., Birdthistle et al., 2018; Harvey et al., 2000).

##### Distribution of evidence across intervention and population

**Figure 20:**
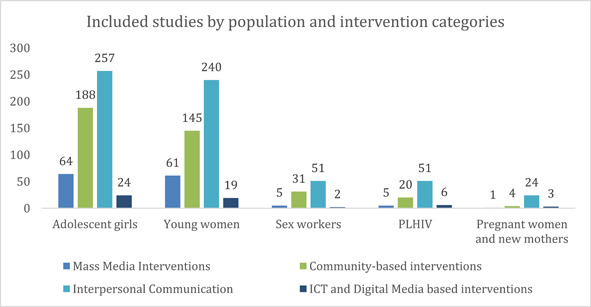
Included studies by population and intervention categories

Most interventions were targeted at adolescent girls (*n* = 533) followed by young women (*n* = 465). However, very few studies focused on population sub‐categories like sex workers (*n* = 89) and PLHIV (*n* = 82). Pregnant women and new mothers (*n* = 32) emerged as one of the least evidenced population sub‐categories. Figure 20 highlights studies across different intervention categories and population sub‐types.

##### Distribution of evidence by intervention and influencer

**Figure 21:**
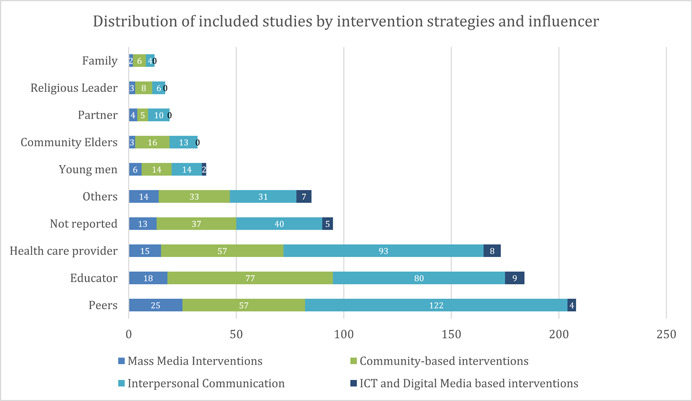
Included studies by influencer and intervention categories

The distribution of included studies across intervention strategies and influencers (Figure 21) suggests that peers (*n* = 208), educators (*n* = 184), and health care providers (*n* = 183) were the most studied influencers. A fewer number of studies included young men (*n* = 36), community elders (*n* = 32), partners (*n* = 19), religious leaders (*n* = 17), and family (*n* = 12).

Interpersonal communication was the most used intervention technique across all sub‐populations, apart from community leaders, religious leaders, and family, where more community‐based interventions were utilized. ICT and digital media‐based tools were most employed for healthcare providers (*n* = 8) and educators (*n* = 9).

##### Distribution of evidence by outcome and population

**Figure 22:**
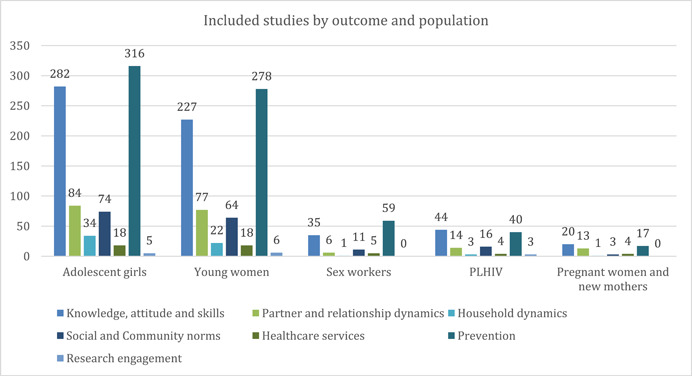
Included studies by outcome category and population

Figure 22 presents studies mapped across outcomes and population categories. While prevention‐related outcomes remain the most dominant category across all population sub‐groups, for pregnant women and new mothers and PHLIV, studies focusing on knowledge attitude and skills are slightly higher. It is interesting to note that no study focused on research engagement‐related outcomes using SBCC tools among sex workers (aged 15–24) despite them being a high‐risk category, and one of the key population groups. The same stands true for pregnant women and new mothers; however, one can understand that despite the at‐risk category, this population subtype is traditionally not engaged in biomedical research.

#### 6.3.3 Quality assessment

**Figure 23:**
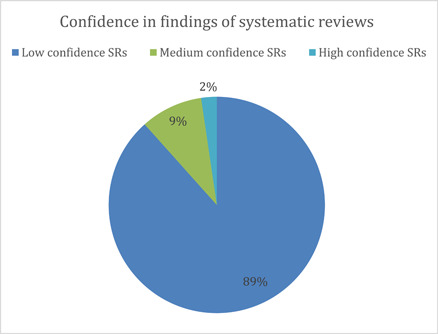
Confidence in findings of systematic reviews

Systematic reviews/meta‐analyses were critically appraised with AMSTAR‐2. A majority of the systematic reviews (89%) are rated as being low confidence (*n* = 38). This was mainly on account of some critical flaws, such as no assessment for risk of bias in primary studies, not using satisfactory techniques for assessing the risk of bias, or not providing the source of funding for the studies included in the review. Of all included systematic reviews, four were found to be of medium confidence (9%) and only one of high confidence (2%) (Figure 23).

#### 6.3.4 Funding agency

**Figure 24:**
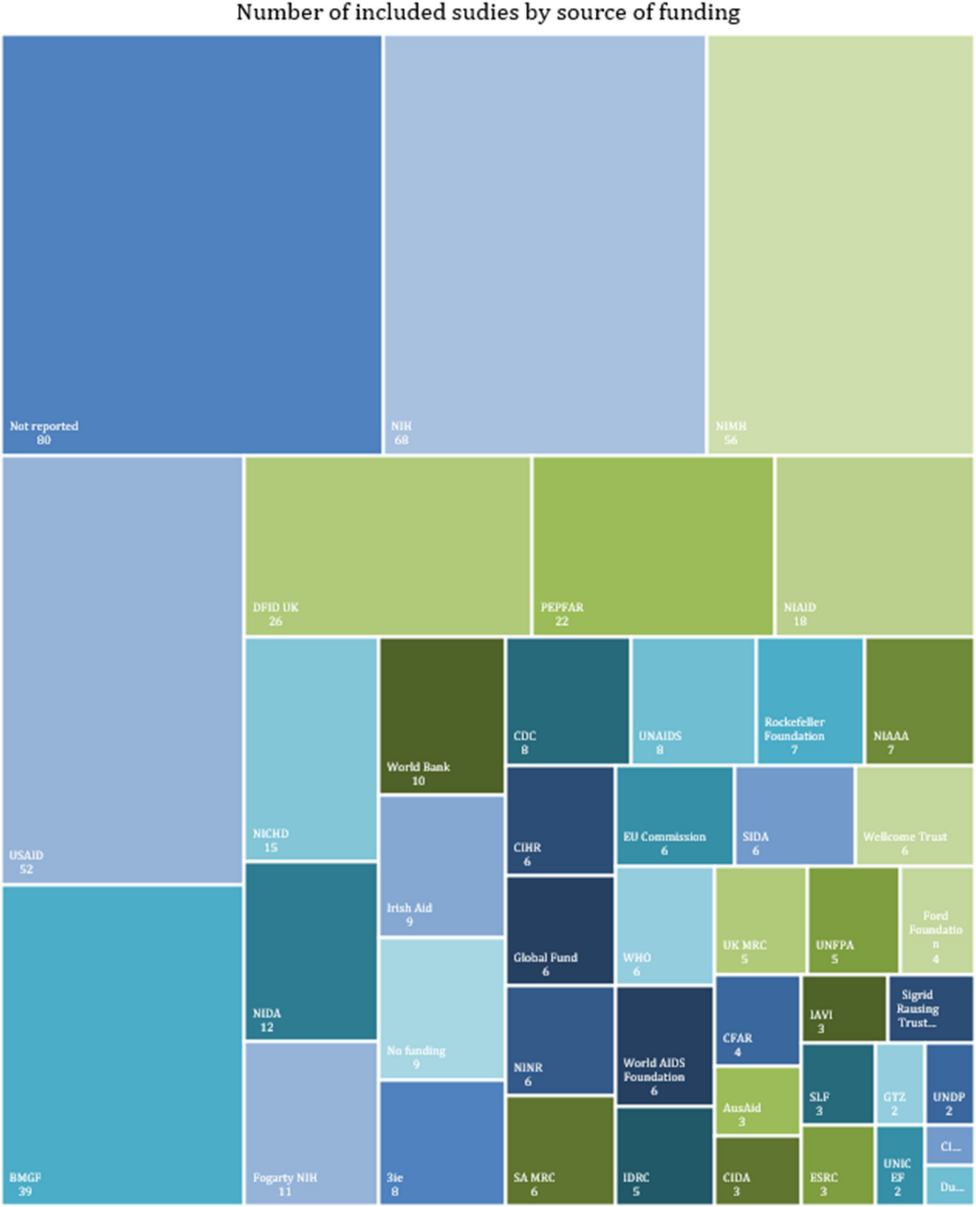
Contribution of funding agencies in the included studies

Most included studies reported (Figure 24) receiving funding from single or multiple sources/bodies. Only 9 included studies reported not having received any funding. As many as 80 studies did not report any source of funding. Some of the major funders include the National Institute of Health (NIH) (*n* = 68), National Institute of Mental Health (NIMH) (*n* = 56), the United States Agency for International Development (USAID) (52), the Bill & Melinda Gates Foundation (BMGF) (*n* = 39), and The Department for International Development (DFID, UK) (*n* = 26). The full list of funding sources is available in Supporting Information: Appendix E.

## 7 DISCUSSION

***Key findings***

This EGM includes studies that employ the use of SBCC interventions targeted towards AGYW and their influencers for HIV prevention and engagement in research. We summarize some of the emerging findings and lessons in this section.

### 7.1 Interventions

Evidence is unevenly distributed across different intervention types. While there is a huge focus on interpersonal communication‐based interventions, followed by community‐based interventions, the use of media and technology‐driven interventions are relatively less studied. These include the use of mass‐media and ICT and digital media‐based interventions.

Despite increased digital penetration of ICT and digital media‐based interventions (Raftree, 2019), evaluations for the same were negligible. While there has been an increased recognition of social media‐based networks to reach hidden at‐risk HIV populations (Pagkas‐Bather et al., 2020), the evaluation of the same to engage AGYW is surprisingly low. Similarly, despite the rapid growth of m‐health services, we see relatively lower number of interventions utilizing mobile‐based services.

Likewise, despite the global recognition of the effectiveness of mass‐media‐based interventions in supporting efforts to reduce the HIV/AIDS burden, the evaluation of these interventions is comparatively lesser. It is important to note here that multiple studies used media‐based interventions along with other intervention types/sub‐types like community‐based interventions and interpersonal communication‐based interventions.

Even within the highly concentrated categories, we see a predominance of certain intervention sub‐types such as peer‐led interactions, facilitated community dialogues and counseling. It is interesting to note that the roll‐out of these interventions among AGYW is heavily dependent on peers, educators, and healthcare providers who are key actors in delivering them. In 26% of included studies (*n* = 120), interventions are delivered by educators, largely in school‐based settings and, in many cases, are part of sex‐education curricula. While the dominant engagement of peers is understandable given they are key influencers within AGYW networks, the lack of engagement of other key influencers such as partners highlights a gap in intervention design. Further, while educators and healthcare providers can play a critical role in knowledge dissemination, their role in creating enabling ecosystems for behavior change is limited without the engagement of key influencers like family, community elders, religious leaders, etc., who can play a direct role in facilitating the uptake of safe and positive sexual health behaviors.

Finally, although community‐based interventions are the second most concentrated area of the map, there are yet gaps in evidence addressing gamification and experiential learning, capacity strengthening, theatre, art‐based approach, and folk media. Gamified learning, in particular, has emerged as one of the most promising interventional approaches to health‐related behavior change (Cugelman, 2013; King et al., 2013). Their unique advantage in terms of both their motivational effect as well as the pedagogical principles they are based on—including user‐centricity, interactivity, repetition, and continuous feedback—make them an especially potent vehicle for health messaging. However, we see that the evidence for these kinds of interventions, both in physical (gamification and experiential learning) and digital spaces (digital games and learning tools), are largely missing.

### 7.2 Outcomes

***Prevention‐related outcomes were most studied. However, outcomes related to more recent methods of prevention were less evidenced***.

Among the outcome categories, prevention‐related outcomes were most measured followed by knowledge, attitude, and skills‐related outcomes. However, within these categories, data was unevenly distributed across sub‐categories. Within prevention‐related outcomes, the most densely evidenced sub‐categories include correct and consistent condom use, limiting sexual partners, and routine testing and status awareness. More recent methods of prevention, such as uptake of PrEP and other biomedical prevention options, were studied less.

***Knowledge related outcomes are most studied with less focus on the continuum of behavior change***.

Behavior change is a dynamic process. At an individual level, the behavior change continuum includes various stages—awareness, knowledge, intent, trial, action, and habit formation (Prochaska & DiClemente, 1983). In a real‐world context, external stimuli shaped by the socio‐cultural realities of an individual continuously affect their behavioral decisions, making this continuum of change highly dynamic in nature. One can understand this as a constant interaction between individuals and their environments (influencers, systems, and social structures) capabilities, opportunities, and motivations (Michie et al., 2014).

The evidence gathered through the EGM highlights that most measured outcomes were towards addressing capability‐related behavioral barriers among AGYW populations and focused mostly on knowledge‐related capabilities such as knowledge and awareness about HIV/STIs and HIV/STI risk perception. Other capability‐related outcomes focusing on the skills of the AGYW or their influencers were relatively less studied. For example, skills that help translate knowledge into real action like negotiation and life skills or provider sensitization and engagement skills were some of the areas with low evidence. With regard to motivational barriers, some were better studied (such as individual agency and self‐efficacy), while life skills, joint decision making in households, etc., were sparsely evidenced. Very few studies measured outcomes addressing aspects of opportunity‐related barriers. These included outcomes related to social and community norms, household dynamics, and health care services, among others.

***Research engagement outcomes remain poorly measured***.

The role of SBCC interventions in supporting research engagement‐related outcomes was poorly evidenced. Of the five studies that measured outcomes related to participation in biomedical research, four were from Sub‐Saharan Africa and the remaining study was from India. Further, only one study measured outcomes related to research awareness and benefit perception.

### 7.3 Others

We find that geographical coverage of evidence remains concentrated in a few Sub‐Saharan African countries, including South Africa, Uganda, Kenya, Tanzania, Zimbabwe, and Nigeria. Subsequently, evidence largely remains unevenly scattered across different regions and countries—in East Asia and the Pacific, evidence remains concentrated within countries like China and Thailand, while in South Asia, India is the only country with a considerable evidence base. There is hardly any evidence from countries like Indonesia, Mozambique, Pakistan, Ukraine, Argentina, Colombia, the Democratic Republic of Congo, etc., despite the high burden of HIV in these countries (UNAIDS, n.d). The MENA region reported the lowest number of studies, which corresponds to the low HIV burden in the region.

There is a clear need for more impact evaluations across many of the intervention and outcome categories, with more even distribution across regions and study design. Currently, RCT‐based studies are mostly done in a few countries in Sub‐Saharan Africa, while China and Thailand are the only countries in East Asia and the Pacific region with RCT‐based studies. In South Asia, India emerges as the only country with RCT‐ based studies (*n* = 5), leaving room for more RCTs and other experimental study designs. There is also an urgent need for better use of high‐quality systematic reviews and implementation research to complement more rigorous evaluations across many components of intervention categories. Currently, most of the systematic reviews included are ranging from low to moderate quality.

### 7.4 Quality of the evidence

The confidence in the findings of systematic reviews was assessed using the AMSTAR‐2 checklist. Most systematic reviews have been rated as being low confidence, followed by medium confidence. Only one systematic review was assessed to be of high confidence, indicating that the findings of these reviews should be used with caution. This was on account of some critical flaws, such as no assessment of risk of bias in primary studies, not using satisfactory techniques for assessing the risk of bias, or not providing the source of funding for the studies included in the review.

### 7.5 Limitations of the EGM

This EGM is based on an as expansive a framework as possible, though limitations may remain in the scope and approach. We trust that other researchers will address the below limitations going forward, contributing to more robust evidence architecture in the future:

1. This EGM assesses only those studies which have been reported or published in the English language. There is, therefore, a likelihood that it misses studies published or reported in other languages, especially from regions where English is not the primary language.
2. While each study was coded by two coders, and in case of conflict a third reviewer was brought in to resolve any disagreement, there were times when arriving at a consensus was challenging as the reviewers did not necessarily read the full text when reviewing coding decisions and worked from notes on the study. Therefore, despite the search strategy being rigorously followed and random quality checks being conducted, there are possibilities of a coding error.
3. While rigorous coding processes were followed and the reviewers conducted snowball checking of references, random quality checks, and held discussions with experts, it is still possible that relevant studies were missed during primary screening or excluded at the title and abstract level stage.
4. Studies with multiple interventions and outcomes appear in multiple quadrants of the map, as has been noted in various tables in this report and, hence, there might be an overlap. Further, randomized controlled trials and other eligible studies included in this map may also be included in systematic reviews, as in most EGMs.
5. Searching the “gray” literature is challenging and there is a possibility that reviewers missed a few studies. However, to strengthen the search, we included limited gray literature on the guidance of the advisory group.
6. Purely qualitative studies were not included in the EGM, thus presenting a limitation that needs attention in future exercises or updates.
7. While the advisory group was consulted at multiple stages, there were areas of disagreement among members. For example, some felt that the EGM should include system‐level interventions, such as financial support for AGYW. We worked with these inputs, but ultimately made judgment calls based on scope, time, and other critical considerations. It is important to note that this EGM has not focused on behavioral interventions that explicitly attempt to address issues around structural and systemic inequities, and interventions that employ strategies outside of the ambit of the SBCC.
8. Finally, cost‐effectiveness data are broadly missing from this evidence base. This is a critical area and would allow policymakers and funders to make better decisions on where to invest in programming and research with limited resources. However, ultimately, this is outside the scope of this EGM.

## 8 AUTHORS CONCLUSIONS

### 8.1 Implications for research, practice, and policy

In this section, we draw linkages to various domains that the EGM touches on and seek to highlight nuances and specific evidence gaps that may not be obvious from the quick snapshots presented above.

We find that, although the evidence base for SBCC interventions is relatively large, it remains unevenly scattered across geographies and population sub‐types and is mostly concentrated across a few interventions and outcomes. Given the high burden of HIV in the Sub‐Saharan Africa region, evidence concentration is understandable. However, other regions with a significant burden of HIV remain less studied, necessitating a more inclusive programming and evidence generation effort that is responsive to the regionally diverse needs of AGYW. Further, differences in quality of reporting make it critical to invest in gathering better quality evidence for the field to inform decision making and program design.

The current landscape of evidence highlights the need for a more nuanced and intersectional approach. It is critical to acknowledge the diversity within AGYW and understand that one size does not fit all. We especially see evidence gaps for certain key sub‐populations, such as pregnant women and new mothers, sex workers, and PLHIV. Also, for the broader AGYW population, aspects of social positionality related to their age, education, marital status, family type (joint/nuclear), of varying sexualities were not explored in the evidence base. Additionally, studies exploring the aspirations, desires, and life and relationship goals of AGYW were found to be largely missing.

All this calls for a behavioral and psychographic segmentation of the AGYW population along with better quality evidence to understand what interventions work, for whom, and towards what outcome. It is important that—going forward—program designers, implementers, and policymakers consider the diversity of this group and better understand behavioral convergences and divergences. There is, therefore, a need to reframe the narrative in a manner that is not solely focused on disease knowledge‐centered outcomes and which suits the needs of the AGYW.

This EGM highlights that evidence is heavily concentrated within the awareness‐intent spectrum of behavior change and gets lean for outcomes situated within the intent‐action and the action‐habit formation spectrum of the behavior change continuum. In line with this, we find that studies mostly measure outcomes around the domains of knowledge, attitude, and skills, but get sparse around pathways enabling change, such as partner and relationship dynamics, household dynamics, and social and community norms, among others. While there is a lot of emphasis on building disease knowledge, improving risk perception, and increasing knowledge about a few prevention options, there is not enough emphasis on developing skills that can help translate intent into action and further habit formation.

We also witness that, within the category of influencers, peers, educators, and healthcare providers were most engaged. While the dominant engagement of peers is understandable given, they are key influencers within AGYW networks, the lack of engagement of other key influencers such as partners highlights a gap in intervention design. Hence, intervention strategies that will create an enabling environment for promoting safe and positive sexual health behaviors are necessary. To achieve this, an ecosystem‐based approach that involves other influencers from their family/community, etc., and creates “safe spaces” for AGYW to openly share and discuss such intimate details of their life can be valuable.

The EGM reveals that most of the SBCC work done in the past is through the medium of interpersonal counseling or community‐based engagements. Despite the penetration and influence of mass media and digital media‐based tools, evidence for the utilization of the same for engaging AGYW remains limited. These media forms have the ability to shape and control “the scale and form of human association and action” (McLuhan & Lapham, 1964), yet remain an untapped opportunity. Given the blurring line between digital and physical spaces, it is important to understand changing media ecologies of AGYW networks and meet them where they are. It is important to be cognizant, however, that while the potential reach of these tools makes these valuable methods of engaging these diverse population, growing concerns around gendered access, issues of privacy, confidentiality, and cyber security warrant the need for more evidence to be generated to better understand and utilize these media forms in appropriate and ethical ways.

While gamified learning has emerged as one of the most promising interventional approaches to health‐related behavior change, the EGM highlights that evidence for these kinds of interventions, both in physical and digital arenas, is under‐explored.

Another key gap, and an urgent intervention area that the EGM identifies, is low evidence of SBCC interventions for outcomes related to research engagement. There is a growing recognition of the need to engage AGYW in biomedical research given the vulnerabilities which put them at higher risk of HIV. However, evidence of interventions that use SBCC strategies for the same is extremely limited. These interventions can play a critical role in helping researchers meaningfully engage with communities by taking their needs into account, collaborate with them as equal partners, and instil a sense of trust and reciprocity. Moving forward, more research is needed to better understand how SBCC tools have been used, towards what end, and what impact they have been able to generate to ensure equitable participation of at‐risk AGYW communities in HIV prevention and biomedical research.

## CONTRIBUTION OF AUTHORS

Content: Devi Leena Bose, Anhad Hundal, Sabina Singh, Kuhika Seth, and Saif ul Hadi.

EGM methods: Ashrita Saran, Jessy Joseph.

Information retrieval: Sabina Singh, Shweta Singh, Anhad Hundal.

## DECLARATIONS OF INTEREST

None.

## PLANS FOR UPDATING EGM

The authors have no immediate plans to update this EGM.

## DIFFERENCES BETWEEN PROTOCOL AND REVIEW

While finalizing this map, there have been some deviations from the protocol, informed by practical considerations, including:

1. Separation of “Community Dialogues” and “Capacity Strengthening” interventions as independent sub‐categories within the larger “Community‐Based Interventions” category.
2. Addition of “Delayed Sexual Debut” within prevention outcome measures.
3. Change from “peer‐peer intervention” to “peer‐led intervention.”
4. Removal of “Intravenous Drug Users,” “STI's” and “Mother‐to‐Child Transmission” as sub‐populations of the key AGYW population group, and inclusion of “Pregnant Women and Young Mothers.”

## SOURCES OF SUPPORT

### 1 Internal sources

No sources of support were provided.

### 2 External sources

The EGM has been supported under the aegis of the ADVANCE (Accelerate the Development of Vaccines and New Technologies to Combat the AIDS Epidemic) program at IAVI, funded by a cooperative agreement through 2026 with the U.S. Agency for International Development (USAID) through the U.S. President's Emergency Plan for AIDS Relief (PEPFAR), aims to work works safe, effective and affordable solutions for HIV.

## Supporting information

Supporting information.

## Footnotes

1 We have included RCTs, as well as other designs, with a comparison group mentioned in the report as impact evaluations. Further, we are aware that not all these designs included as impact evaluations, if assessed from a risk of bias tool, will indicate the same level of confidence as we can put in experimental designs as RCTs.
