# Corrigendum to “Evidence and gap map report: Social and Behavior Change Communication (SBCC) interventions for strengthening HIV prevention and research among adolescent girls and young women (AGYW) in low‐ and middle‐income countries (LMICs)”

**DOI:** 10.1002/cl2.1310

**Published:** 2023-02-12

The Plain Language Summary published with above EGM report should correctly read as:

**There is scattered evidence on the effectiveness of social and behaviour change communication interventions for HIV prevention among adolescent girls and young women**.

Interest in socio‐behavioural interventions to support demand and uptake of HIV services is on the rise. Therefore, the evidence of their effectiveness needs to be systematically mapped to enable informed decision‐making by sponsors, researchers, policymakers and public health experts.

This evidence and gap map (EGM) presents a consolidated evidence base of existing social and behaviour change communication interventions, and the end‐user behaviours they target, in low‐ and middle‐income countries. It looks at a range of strategies including mass media, community‐based interventions, interpersonal interventions, and ICT and digital media‐based interventions, and identifies gaps in programming.
