# Supplementary material for: Evidence and gap map report: Social and Behavior Change Communication (SBCC) interventions for strengthening HIV prevention and research among adolescent girls and young women (AGYW) in low‐ and middle‐income countries (LMICs)
Source: Campbell Syst Rev. 2023 Jan 10;19(1):e1297. doi: 10.1002/cl2.1297 (PMC9831290; doi:10.1002/cl2.1297)
Supplement: Supplementary file 1 — Supporting information. [file CL2-19-e1297-s001.docx]

# **Appendices**

**Appendix A: Search Strategy**

1. **Ovid MEDLINE(R) and Epub Ahead of Print, In-Process & Other Non-Indexed Citations, Daily and Versions(R) <1946 to January 19, 2021> 21^st^ January 2021**

1 (HIV* or "human immunodeficiency virus" or "AIDS" or "acquired immunodeficiency syndrome").ti,ab,kw. (422211)

2 exp HIV Infections/pc, px, tm [Prevention & Control, Psychology, Transmission] (88273)

3 1 or 2 (432151)

4 Behavioral Research/ (3344)

5 adolescent behavior/ or behavior therapy/ or information seeking behavior/ or negotiating/ or exploratory behavior/ or health behavior/ or health risk behaviors/ or contraception behavior/ or risk reduction behavior/ or hiv serosorting/ or risk-taking/ or sexual behavior/ or exp coitus/ or courtship/ or safe sex/ or sexual abstinence/ or sexual harassment/ or unsafe sex/ (227187)

6 (behavio* or (information adj2 seek*) or ((abstenen* or abstain* or harass* or safe or unsafe) adj sex*) or "Social and Behavior Change Communication").ti,ab,kw. (1301489)

7 or/4-6 (1422708)

8 preventive health services/ or early intervention, educational/ or primary prevention/ (35372)

9 (prevent* or avoid* or evad* or evasion or abstain* or abstinence or refrain*).ti,ab,kw. (1885590)

10 or/8-9 (1899743)

11 (Intervention or promot* or initiative* or (behavio?r adj change) or (health adj education) or strateg* or television or tv or radio or narrative* or mhealth or ehealth or "text messag*" or edutainment or "mid media" or "mass media" or "street play" or "community mobili?ation" or "mobile health" or "health literacy" or "motivational interview*" or "folk danc*" or "traditional media" or poster* or pamphlet* or leaflets or "information education communication" or IEC or "knowledge attitude and practice" or (communication adj2 channel*) or "care group*" or "community outreach" or "primary health care" or "referral level facilit*" or "task shifting" or "health planning" or "information services" or "participatory learning" or "home visit*" or "inter personal communication" or "interpersonal communication" or SBCC or "audio messag*" or "video messag*" or book or books or helpline or pictures or "animation video*").ti,ab,kw. (3246451)

12 health education/ or consumer health information/ or health literacy/ or health promotion/ or sex education/ or teach-back communication/ or communication/ or information seeking behavior/ or mass media/ or radio/ or television/ or videodisc recording/ or videotape recording/ or publications/ or books/ or pamphlets/ or newspapers as topic/ or periodicals as topic/ or teaching materials/ or audiovisual aids/ or multimedia/ or telemedicine/ or cell phone/ or text messaging/ or Health Knowledge, Attitudes, Practice/ or community networks/ or community participation/ or Community-Institutional Relations/ or Primary Health Care/ or "Referral and Consultation"/ or Regional Health Planning/ or Community Health Planning/ or Strategic Planning/ or access to information/ or communication barriers/ or health communication/ or information dissemination/ or information literacy/ or persuasive communication/ or information services/ or house calls/ or verbal behavior/ or speech/ or speech intelligibility/ or call centers/ or hotlines/ (699871)

13 or/11-12 (3739625)

14 adolescent/ or young adult/ (2471922)

15 female/ or women/ (8893119)

16 14 and 15 (1956198)

17 (((woman* or women* or girl* or female* or gender) adj2 (adolescen* or "young adult" or teen* or "emerging adult" or "young person*" or "young people" or juvenile* or minor)) or schoolgirl*).ti,ab,kw. (31528)

18 16 or 17 (1966035)

19 Randomized Controlled Trial/ or Randomized Controlled Trials as Topic/ or random allocation/ or Propensity Score/ or Quasi-Experimental Studies/ (751730)

20 Program Evaluation/ or Evaluation Studies/ (312045)

21 ((impact adj2 (evaluat* or assess* or analy* or estimat* or measure)) or (effectiveness adj2 (evaluat* or assess* or analy* or estimat* or measure))).ti,ab,kw. (181245)

22 ("program* evaluation" or "project evaluation" or "evaluation research" or "natural experiment*" or "program* effectiveness").ti,ab,kw. (13119)

23 systematic review/ or meta analysis/ (208378)

24 controlled before-after studies/ or cross-sectional studies/ or interrupted time series analysis/ or qualitative research/ (409288)

25 (random* or experiment* or (match* adj2 (propensity or coarsened or covariate)) or "propensity score" or "difference in difference*" or "difference-in-difference*" or "differences in difference*" or "differences-in-difference*" or "double difference*" or "quasi-experimental" or "quasi experimental" or "quasi-experiment" or "quasi experiment" or ((estimator or counterfactual) and evaluation*) or "instrumental variable*" or (IV adj2 (estimation or approach)) or "regression discontinuity" or "time series" or "segment* regression" or (non adj2 participant*) or ((control or comparison) adj2 (group* or condition* or area* or intervention))).ti,ab,kw. (3685804)

26 ("quasi experiment*" or quasi-experiment* or "random* control* trial*" or "random* trial*" or rct* or (random* adj3 allocat*) or evaluat* or impact* or assess* or dif-dif or psm or "double difference" or difference-in-difference or rdd or "difference in difference" or "statistical matching*" or "propensity score matching" or "covariate matching" or "coarsened-exact matching" or "propensity-weighted" or "multiple regression" or "statistical regression" or "regression discontinuity*" or "cohort analysis" or "quantitative method*" or "program* evaluation" or "interrupted time series" or (before adj5 after) or (pre adj5 post) or ((pretest or "pre test") and (posttest or "post test")) or (("fixed effect*" or "random effect*") adj3 (model or estimation)) or "instrumental variable" or "synthetic control" or ((quantitative or qualitative or "comparison group*" or counterfactual or "counter factual" or counter-factual or experiment* or panel or cross-sectional) adj3 (design or study or analysis))).ti,ab,kw. (7290257)

27 ((Systematic* or synthes*) adj3 (Research or evaluation* or finding* or thematic* or report or descriptive or explanatory or narrative or meta* or review* or data or literature or studies or evidence or map or quantitative or study or studies or paper or impact or impacts or effect* or compar*)).ti,ab,kw. (388128)

28 ("Meta regression" or "meta synth*" or "meta-synth*" or "meta analy*" or "metaanaly*" or "meta-analy*" or "metanaly*" or "Metaregression" or "Meta-regression" or "Methodologic* overview" or "pool* analys*" or "pool* data" or "Quantitative* overview" or "research integration").ti,ab,kw. (210420)

29 (review adj3 (effectiveness or effects or systemat* or synth* or integrat* or map* or methodologic* or quantitative or qualitative or evidence or literature)).ti,ab,kw. (471205)

30 or/19-29 (10056658)

31 (afghanistan or albania or algeria or american samoa or angola or "antigua and barbuda" or antigua or barbuda or argentina or armenia or armenian or aruba or azerbaijan or bahrain or bangladesh or barbados or republic of belarus or belarus or byelarus or belorussia or byelorussian or belize or british honduras or benin or dahomey or bhutan or bolivia or "bosnia and herzegovina" or bosnia or herzegovina or botswana or bechuanaland or brazil or brasil or bulgaria or burkina faso or burkina fasso or upper volta or burundi or urundi or cabo verde or cape verde or cambodia or kampuchea or khmer republic or cameroon or cameron or cameroun or central african republic or ubangi shari or chad or chile or china or colombia or comoros or comoro islands or iles comores or mayotte or democratic republic of the congo or democratic republic congo or congo or zaire or costa rica or "cote d’ivoire" or "cote d’ ivoire" or cote divoire or cote d ivoire or ivory coast or croatia or cuba or cyprus or czech republic or czechoslovakia or djibouti or french somaliland or dominica or dominican republic or ecuador or egypt or united arab republic or el salvador or equatorial guinea or spanish guinea or eritrea or estonia or eswatini or swaziland or ethiopia or fiji or gabon or gabonese republic or gambia or "georgia (republic)" or georgian or ghana or gold coast or gibraltar or greece or grenada or guam or guatemala or guinea or guinea bissau or guyana or british guiana or haiti or hispaniola or honduras or hungary or india or indonesia or timor or iran or iraq or isle of man or jamaica or jordan or kazakhstan or kazakh or kenya or "democratic people’s republic of korea" or republic of korea or north korea or south korea or korea or kosovo or kyrgyzstan or kirghizia or kirgizstan or kyrgyz republic or kirghiz or laos or lao pdr or "lao people's democratic republic" or latvia or lebanon or lebanese republic or lesotho or basutoland or liberia or libya or libyan arab jamahiriya or lithuania or macau or macao or republic of north macedonia or macedonia or madagascar or malagasy republic or malawi or nyasaland or malaysia or malay federation or malaya federation or maldives or indian ocean islands or indian ocean or mali or malta or micronesia or federated states of micronesia or kiribati or marshall islands or nauru or northern mariana islands or palau or tuvalu or mauritania or mauritius or mexico or moldova or moldovian or mongolia or montenegro or morocco or ifni or mozambique or portuguese east africa or myanmar or burma or namibia or nepal or netherlands antilles or nicaragua or niger or nigeria or oman or muscat or pakistan or panama or papua new guinea or new guinea or paraguay or peru or philippines or philipines or phillipines or phillippines or poland or "polish people's republic" or portugal or portuguese republic or puerto rico or romania or russia or russian federation or ussr or soviet union or union of soviet socialist republics or rwanda or ruanda or samoa or pacific islands or polynesia or samoan islands or navigator island or navigator islands or "sao tome and principe" or saudi arabia or senegal or serbia or seychelles or sierra leone or slovakia or slovak republic or slovenia or melanesia or solomon island or solomon islands or norfolk island or norfolk islands or somalia or south africa or south sudan or sri lanka or ceylon or "saint kitts and nevis" or "st. kitts and nevis" or saint lucia or "st. lucia" or "saint vincent and the grenadines" or saint vincent or "st. vincent" or grenadines or sudan or suriname or surinam or dutch guiana or netherlands guiana or syria or syrian arab republic or tajikistan or tadjikistan or tadzhikistan or tadzhik or tanzania or tanganyika or thailand or siam or timor leste or east timor or togo or togolese republic or tonga or "trinidad and tobago" or trinidad or tobago or tunisia or turkey or turkmenistan or turkmen or uganda or ukraine or uruguay or uzbekistan or uzbek or vanuatu or new hebrides or venezuela or vietnam or viet nam or middle east or west bank or gaza or palestine or yemen or yugoslavia or zambia or zimbabwe or northern rhodesia or global south or africa south of the sahara or sub-saharan africa or subsaharan africa or africa, central or central africa or africa, northern or north africa or northern africa or magreb or maghrib or sahara or africa, southern or southern africa or africa, eastern or east africa or eastern africa or africa, western or west africa or western africa or west indies or indian ocean islands or caribbean or central america or latin america or "south and central america" or south america or asia, central or central asia or asia, northern or north asia or northern asia or asia, southeastern or southeastern asia or south eastern asia or southeast asia or south east asia or asia, western or western asia or europe, eastern or east europe or eastern europe or developing country or developing countries or developing nation? or developing population? or developing world or less developed countr* or less developed nation? or less developed population? or less developed world or lesser developed countr* or lesser developed nation? or lesser developed population? or lesser developed world or under developed countr* or under developed nation? or under developed population? or under developed world or underdeveloped countr* or underdeveloped nation? or underdeveloped population? or underdeveloped world or middle income countr* or middle income nation? or middle income population? or low income countr* or low income nation? or low income population? or lower income countr* or lower income nation? or lower income population? or underserved countr* or underserved nation? or underserved population? or underserved world or under served countr* or under served nation? or under served population? or under served world or deprived countr* or deprived nation? or deprived population? or deprived world or poor countr* or poor nation? or poor population? or poor world or poorer countr* or poorer nation? or poorer population? or poorer world or developing econom* or less developed econom* or lesser developed econom* or under developed econom* or underdeveloped econom* or middle income econom* or low income econom* or lower income econom* or low gdp or low gnp or low gross domestic or low gross national or lower gdp or lower gnp or lower gross domestic or lower gross national or lmic or lmics or third world or lami countr* or transitional countr* or emerging economies or emerging nation?).ti,ab,sh,kf. (2005406)

32 3 and 7 and 10 and 13 and 18 and 30 and 31 (1859)

33 limit 32 to (english language and yr="2000 -Current") (**1673**)

**Appendix B: Included studies by country and study design**

| Country | Randomized Controlled Trial | Quasi-Experimental | Cross-Sectional or Panel Studies | Systematic Review | Process Evaluation | Total |
| --- | --- | --- | --- | --- | --- | --- |
| Armenia | 1 | 0 | 0 | 0 | 0 | 1 |
| Argentina | 0 | 0 | 0 | 1 | 0 | 1 |
| Bangladesh | 0 | 1 | 1 | 4 | 1 | 6 |
| Belize | 1 | 1 | 0 | 3 | 0 | 5 |
| Benin | 1 | 1 | 0 | 0 | 0 | 2 |
| Brazil | 0 | 2 | 4 | 6 | 0 | 12 |
| Botswana | 1 | 2 | 2 | 6 | 1 | 11 |
| Burkina Faso | 0 | 1 | 1 | 5 | 0 | 7 |
| Cambodia | 1 | 0 | 1 | 0 | 0 | 2 |
| Cameroon | 0 | 3 | 1 | 9 | 0 | 13 |
| Chile | 0 | 0 | 0 | 5 | 0 | 5 |
| China | 10 | 12 | 6 | 8 | 3 | 36 |
| Congo | 0 | 1 | 3 | 3 | 0 | 7 |
| Colombia | 0 | 1 | 2 | 4 | 1 | 7 |
| Dominican Republic | 0 | 1 | 0 | 3 | 0 | 4 |
| Ethiopia | 1 | 4 | 4 | 6 | 1 | 15 |
| El Salvador | 0 | 1 | 0 | 0 | 0 | 1 |
| Gabon | 0 | 0 | 0 | 2 | 0 | 2 |
| Gambia | 0 | 0 | 0 | 1 | 0 | 1 |
| Gaza | 0 | 0 | 0 | 1 | 0 | 1 |
| Ghana | 0 | 1 | 3 | 11 | 0 | 15 |
| Guatemala | 0 | 1 | 1 | 1 | 0 | 3 |
| Guinea-Bissau | 0 | 2 | 0 | 3 | 1 | 5 |
| Haiti | 0 | 1 | 1 | 1 | 0 | 3 |
| India | 5 | 12 | 20 | 12 | 0 | 49 |
| Indonesia | 0 | 1 | 0 | 1 | 0 | 2 |
| Iran | 2 | 2 | 0 | 1 | 0 | 5 |
| Israel | 0 | 0 | 0 | 1 | 0 | 1 |
| Ivory Coast | 0 | 1 | 1 | 3 | 0 | 5 |
| Kazakhstan | 1 | 0 | 0 | 0 | 0 | 1 |
| Kenya | 13 | 9 | 14 | 21 | 0 | 57 |
| Jamaica | 2 | 0 | 1 | 2 | 0 | 5 |
| Lebanon | 0 | 0 | 0 | 1 | 0 | 1 |
| Lesotho | 0 | 0 | 0 | 1 | 0 | 1 |
| Liberia | 1 | 1 | 0 | 4 | 0 | 6 |
| Malawi | 4 | 4 | 1 | 9 | 1 | 18 |
| Madagascar | 0 | 0 | 0 | 4 | 0 | 4 |
| Malaysia | 0 | 1 | 0 | 1 | 0 | 2 |
| Mexico | 5 | 3 | 1 | 6 | 0 | 15 |
| Mozambique | 0 | 1 | 4 | 4 | 1 | 9 |
| Mongolia | 0 | 1 | 0 | 2 | 0 | 3 |
| Myanmar (Burma) | 1 | 0 | 1 | 1 | 0 | 3 |
| Namibia | 2 | 1 | 0 | 9 | 0 | 12 |
| Nepal | 0 | 2 | 3 | 3 | 0 | 8 |
| Nicaragua | 0 | 0 | 0 | 4 | 0 | 4 |
| Niger | 0 | 0 | 0 | 1 | 0 | 1 |
| Nigeria | 6 | 10 | 6 | 15 | 1 | 37 |
| Pakistan | 0 | 0 | 1 | 2 | 0 | 3 |
| Peru | 2 | 1 | 0 | 6 | 0 | 9 |
| Philippines | 0 | 2 | 0 | 6 | 0 | 8 |
| Russia | 3 | 1 | 1 | 3 | 0 | 8 |
| Rwanda | 0 | 1 | 4 | 5 | 0 | 10 |
| Senegal | 0 | 2 | 1 | 2 | 0 | 5 |
| Sierra Leone | 0 | 0 | 0 | 2 | 0 | 2 |
| South Africa | 35 | 28 | 18 | 27 | 8 | 107 |
| Sri Lanka | 0 | 0 | 0 | 2 | 0 | 2 |
| South Sudan | 0 | 0 | 0 | 1 | 0 | 1 |
| St Lucia | 0 | 0 | 0 | 3 | 0 | 3 |
| St. Vincent and the Grenadines | 0 | 0 | 0 | 1 | 0 | 1 |
| Suriname | 0 | 1 | 0 | 0 | 0 | 1 |
| Swaziland | 2 | 1 | 0 | 1 | 0 | 4 |
| Thailand | 4 | 5 | 4 | 11 | 0 | 24 |
| Tanzania | 8 | 8 | 8 | 22 | 0 | 46 |
| Uganda | 20 | 7 | 9 | 25 | 2 | 61 |
| Ukraine | 0 | 1 | 0 | 2 | 0 | 3 |
| Turkey | 1 | 0 | 0 | 1 | 0 | 2 |
| Vietnam | 2 | 1 | 2 | 2 | 0 | 7 |
| Zambia | 9 | 4 | 2 | 15 | 1 | 30 |
| Zimbabwe | 10 | 4 | 8 | 21 | 2 | 43 |
| Not reported | 0 | 0 | 0 | 4 | 0 | 4 |
| **Total** | **137** | **144** | **135** | **43** | **24** | **458** |

**Appendix C: Advisory Group**

| **Name** | **Area of Expertise** | **Organization** |
| --- | --- | --- |
| Dr. Anant Bhan | Global Health, Bioethics | Independent, Ex-President, International Association of Bioethics |
| Dr Venkatesh Chakrapani | HIV/AIDS and sexual health issues of sexual minorities | Founder and Director, C-SHaRP |
| Ms. Shruta Rawat | Gender and sexual minorities. | Research Manager, Humsafar Trust |
| Dr. Seema Sahay | HIV/AIDS Anthropology and Good Participatory Practices in Research | Scientist G, ICMR National AIDS Research Institute (NARI), Government of India |
| Dr. Aparna Khanna | Development Communication, ICT, and Game design. | Associate Professor, Department of Development Communication and Extension, Lady Irwin College, Delhi University |
| Dr. Manjulika Vaz | Health Humanities, Participatory Research | Lecturer, St. John’s Research Institute |
| Dr. Tapati Dutta | Health education and Health Behavior | Assistant Professor, Fort Lewis College |
| Manju Chatani Gada | AGYW, SRHR, Gender | AVAC |
| Hemlata Verma | Gender mainstreaming, Gender-Based Violence (GBV) | International Centre for Research on Women (ICRW) |
| Renu Golwalkar | Gender Equity, youth, social inclusion | Engender Health |

**Appendix D: Screening Tool**

1. Is the paper published in or beyond 2000?

2. Is the paper in English?

3. Is the paper about an intervention intended to modify the behavior or attitudes, either directly or indirectly, of Adolescent Girls and Young Women (age 15-24) or their parents, partners, peers, community influencers like religious leaders, community elders, professionals with whom they interact?

4. Is the paper-based in a low- and middle-income country/countries?

5. Is the paper a quantitative evaluation reporting measures of eligible outcomes compared to the outcomes (1) in a comparison group (either with or without baseline outcome measures), (2) before versus after with no comparison group, or (3) a systematic review** of such studies?

6. Do the outcomes include measures of attitudes, beliefs, or behavior of AGYW, their influencers, and partners or professionals?

**Appendix E: Coding Tool**

- Region
  - East Asia and Pacific
  - Europe and Central Asia
  - Latin America and Carribean
  - Middle East and North Africa
  - South Asia
  - Sub-Saharan Africa
  - Not Reported
- Country
  - Afghanistan
  - Angola
  - Armenia
  - Argentina
  - Bahrain
  - Bangladesh
  - Barbados
  - Belarus
  - Belize
  - Bolivia
  - Brazil
  - Botswana
  - Bulgaria
  - Burkina Faso
  - Cambodia
  - Cameroon
  - Chile
  - China
     *If just Hong Kong, use Hong King code only, NOT China*
  - Congo
  - Croatia
  - Cuba
  - Colombia
  - Cyprus
  - Dominican Republic
  - Egypt
  - Ecuador
  - Eritrea
  - Estonia
  - Finland
  - Ethiopia
  - Gambia, The
  - Georgia
  - Ghana
  - Greece
  - Guatemala
  - Guinea-Bissau
  - Haiti
  - Honduras
  - Hong Kong
  - India
  - Indonesia
  - Iran
  - Ireland
  - Israel
  - Italy
  - Ivory Coast
  - Kazakhstan
  - Jordan
  - Kenya
  - Korea
  - Kuwait
  - Japan
  - Jamaica
  - Lao
  - Latin America (where multiple countries)
  - Lebanon
  - Lesotho
  - Liberia
  - Latvia
  - Lithuania
  - Luxembourg
  - Malawi
  - Madagascar
  - Malaysia
  - Mali
  - Mexico
  - Micronesia
  - Marshall Islands
  - Mozambique
  - Mongolia
  - Myanmar (Burma)
  - Namibia
  - Nepal
  - New Zealand
  - The Netherlands
  - Nicaragua
  - Niger
  - Nigeria
  - Northern Ireland
  - Pakistan
  - Norway
  - Panama
  - Papua New Guinea
  - Peru
  - Philippines
  - Poland
  - Portugal
  - Puerto Rico
  - Romania
  - Russia
  - Rwanda
  - Samoa
  - San Marino
  - Saudi Arabia
  - Scotland
  - Serbia
  - Senegal
  - Singapore
  - Sierra Leone
  - Slovakia
  - Slovenia
  - South Africa
  - Spain
  - Sri Lanka
  - Sudan
  - South Sudan
  - St Lucia
  - Swaziland
  - Syria
  - Taiwan
  - Thailand
  - Tanzania
  - Tunisia
  - Uganda
  - Ukraine
  - Turkey
  - Uzbekistan
  - Vanuatu
  - Venezuela
  - Vietnam
  - West Indies
  - Yemen
  - Zambia
  - Zimbabwe
  - St. Vincent and the Grenadines
  - Benin
  - Gabon
  - Suriname
  - El Salvador
  - Gaza
  - Not reported
- Setting
  - Rural
  - Urban
  - Formal
  - Informal
- Population
  - Adolescent girls
  - Young women
  - Sex workers
  - PLHIV
  - Pregnant women and new mothers
  - Others
- Age group
  - 15-19
  - 20-24
  - Not reported
- Influencers
  - Young men
  - Family
  - Partner
  - Educator
  - Religious Leader
  - Peers
  - Community Elders
  - Health care provider
  - Not reported
  - Others
- Study Design
  - Randomized Controlled Trial
  - Quasi-Experimental
  - Cross-Sectional or Panel Studies
     *with an intervention and comparison group using method to control for selection bias and confounding*
  - Systematic Review
  - Process Evaluation
- Publication Status
  - Published
  - Ongoing
- Intervention strategies
  - Mass Media Interventions
    - Print Media
    - Electronic Media
  - Community-based interventions
     *community based, community owned, participatory, community led, community supported*
    - Community Media
       *Community radio, Local television, community video, community newspaper, newsletter, participatory radio, community screening, community supported media, local radio, digital storytelling, community photography, community storytelling*
    - Folk Media
       *folk media, folklore, folktales, traditional media, lore, legend, fable, myth, culture, tale, oral tradition, oral history, storytelling, oral stories,*
    - Theatre and arts-based approaches
       *Street theatre, performance, performance art, dramatic art, stage, acting, performing, dramatics, show, entertainment, tradition, culture, story-telling, performative arts, dance theatre, participatory theatre, theatre of the oppressed, dance-based, music-based, drama*
    - Community Dialogues
    - Capacity Strengthening
    - Gamification and Experiential Learning
       *Learning, immersive learning, simulated learning, observation, experimental, art, painting, performance art, fine art, arts and crafts, participatory theatre, experiential learning, protests, rally, assembly, clowning, miming, arts-based public health engagement*
  - Interpersonal Communication
     *Interindividual, inter-social, person-to-person, individual, mutual, communicative*
    - Counselling (One-on-One/ Couple Counselling)
    - Home Visits
       *Door-to-door, survey, house-to-house, canvass, direct, study, observe*
    - Peer-led intervention
       *peer conversations, discussions, dialogue, debates, meetings, interviews, peer support groups,*
  - ICT and Digital Media based interventions
     *Electronic media, online media, digital information, data, database, media asset, electronic medium, computerized, online version, electronic mass media, digital multimedia, electronic press*
    - Social Media
       *Social network, Facebook, Instagram, Twitter, Snapchat, Whatsapp, Youtube, TikTok, chat rooms, message boards, communication, message, Pinterest, Google Plus, WeChat, Telegram, web-based, apps, applications, download services, blogs, forums, photo sharing, video sharing, platform*
    - Mobile-Based Services
       *Information technology, web, voice mail, SMS, MMS, text, message, text message, IVRS, internet, e-mail, online mail, electronic mail, message, communication, platform*
    - Digital Games and Learning Tools
       *Games, electronic games, in-app games, in-app advertisements, virtual learning, e-learning, program, computer program, software*
    - Interactive App-Based Services
       *Hands-on, interactive, responsive, reciprocal, online, application, computer program*
- Outcomes
  - Knowledge, attitude, and skills
    - Knowledge and awareness about HIV/STI
       *Awareness, education, health knowledge, health understanding, learning, cognition, science, familiarity, grasp, expertise, know-how, perception, understanding, recognition, literacy, schooling, experience, insight, proficiency, comprehension, realization, attention, observation, HIV status, HIV testing, infection, risk of infection, mother-to-child transmission, sexual transmission, drug use, drug abuse, a person living with HIV (PLHIV), screening, diagnosis, treatment, ART, medicine*
    - HIV/STI risk perception
       *Perception, understanding, judgment, sensitivity, consciousness, insight, conception, notion, thought, recognition, concept, viewpoint, discrimination, stigma, social perception, social stigma, risk, risk-taking, riskbehaviorss, risk-reduction behaviors, attitude, idea, apprehension*
    - Trust in healthcare providers/services
    - Individual agency and self-efficacy
       *Confidence, self-confidence, faith, reliance, belief, care, dependence, obligation, responsibility, presumption, expectation, self-assurance, self-reliance, self-belief, self-motivation, accountability, liability, burden, duty, commitment, requirement*
    - Negotiation and life skills
       *Discussion, discourse, dialogue, debate, agreement, mediation, brokering, consultation, settlement, intervention, meeting, bargain, understanding, conciliation, daily life, day-to-day life, practical knowledge, practical skills, practical competence, life experience, elementary knowledge*
  - Partner and relationship dynamics
     *Response, understanding, partnership, bond, kinship, love, involvement, interdependence, dependence, communication, interrelationship, marriage, relation, spouse, relationship, relationship-building, family dynamics, household dynamics*
    - Partner's HIV/STI awareness
       *Awareness, education, health knowledge, health understanding, learning, cognition, science, familiarity, grasp, expertise, know-how, perception, understanding, recognition, literacy, schooling, experience, insight, proficiency, comprehension, realization, attention, observation, testing, HIV testing, HIV status, infection, risk of infection, contraception, condom, mother-to-child transmission, prevention, messaging, sexual values, sexual transmission, unsafe sexual practices, drug use, drug abuse, a person living with HIV (PLHIV), screening, diagnosis, treatment, ART, medicine*
    - Power equity and role in decision making
       *Equality, fairness, gender equity, gendered roles, household decision making,decision-makerr, head of household, breadwinner, head, authority, patriarchal, patriarch, integrity, justness, right, honesty, investment, fair-mindedness, honor, share, stake, stakeholder, involvement, involve, participation, participate, active participation, passive participation, mobility, relationship*
    - Sexual and intimate partner violence
       *Domestic violence, domestic abuse, domestic assault, abuse, physical abuse, mental abuse verbal abuse, verbal assault, conjugal violence, family violence, violence, force, spousal violence, spousal abuse, intra-family violence, rape, molestation, marital rape, sexual assault, sexual harassment, sexual misconduct, sexual coercion, coercion, intimidation, aggressive behavior, relationship abuse, manipulation, controlling, violence against women, violence against children, child sexual abuse, sex crime, gender-specific violence*
  - Household dynamics
     *Family, relationships, intra-household, parents, in-laws, mother-in-law, father-in-law, sister-in-law, brother-in-law, cousins, extended family, parentage, siblings, sister, brother, mother, father, marriage, partner, husband, spouse, loved ones, children, child, kin, relatives, relations, grandmother, grandfather, grandparents, aunt, uncle, next of kin, home, family unit, joint family, nuclear family, tradition, traditional, customs, values, contact, communication, interaction, interrelation, interrelational, cooperation, reciprocal, interdependent, dependent, dependents, head of household, patriarchal, patriarch, head of family, household head, decision-maker, breadwinner, earner*
    - Parent/in-law communication
       *Mother, father, mother-in-law, father-in-law, parents-in-law, kin, family, conversation, advice, guidance, help, judgment, discussion, recommendation, suggestion, disclose, disclosing, protective, protection, support, fears, facilitators, barriers, communication, sexual communication, stigma, discrimination*
    - Joint decision making in households
       *Joint family, nuclear family, family, head of household, household head, breadwinner, earner, finances, financial stability, children, child, schooling, education, literacy, resolve, take action, judgment making, choice-making, choices, traditions, customs*
  - Social and Community norms
    - Gender norms and expectations
       *Gender, gendered, masculinity, femininity, status-quo, stigma, discrimination, gender roles, role distribution, stereotypes, stereotypical gender roles, gender stereotypes, tradition, values, customs, cultural norms, sexual roles, sexism, division of labor, delineation of roles, misogyny, gender-specific, behavioral norms, division of responsibilities, assigning roles, allocation of duties,*
    - HIV/STI myths and misperceptions
       *Misconceptions, misrepresentations, false notions, stereotypes, stereotyping preconceptions, popular beliefs, public perceptions, public opinion, prejudices, fiction, fabrication, assumptions, traditional thinking, transmission, spread, propagation*
    - Stigma and discrimination
       *Prejudice, prejudicial, judgment, perception, misconception, bigotry, inequality, inequity, gender norms, sexism, discriminatory practices, narrow-mindedness, unequal treatment, mistreatment, disparate practices*
    - Community support systems
       *Aid systems, welfare, welfare schemes, community services, self-help groups, therapy, counseling, safety net, friends, peers, family, extended family, support group, support network, network, assistance systems, helpers, aides*
  - Healthcare services
     *Health service, frontline health workers, frontline workers, health workers, healthcare aides, medical facilities, health facility, psychiatric facilities, health-care, healthcare provisions, medical services, healthcare providers, healthcare professionals, doctors, nurses, psychiatrists, counselors, therapists, healthcare organizations, assistance providers, medical providers, healthcare workforce, health staff, staff, practitioners, scientists, researchers, health personnel, caregivers, hospitals, health utilities, relief workers, healthcare centers, clinic, dispensary, healthcare institutions, medical facility, facility, government hospital, private hospital, treatment facility, public sector, private sector*
    - Provider sensitization and engagement skills
       *Raising awareness, awareness-raising, sensitize, make aware, outreach, outreach efforts, advocate, advocacy, educate, teach, awareness-building, informing, promote, communication, provide knowledge, information, enhancing awareness, understanding, better understanding, recognition, involvement, listening, empathy, trust, interpersonal skills, peer-peer communication, effective communication*
    - Quality of care/satisfaction with services
       *Quality of service, quality of healthcare, quality of health services, quality of aid, quality of services provided, degree of care, standard of care, quality of provision, level of service, gratification, happiness, appreciation, personal satisfaction, patient satisfaction*
  - Prevention
     *Preventive, preventive measures, prophylaxis, deterrence, prevention measures, control*
    - Correct & Consistent condom use
       *Contraception, birth control, condoms, contraception method, pregnancy prevention methods, family planning, contraceptive precautions, prophylaxis, IUD, birth control pills, abstinence, safe sex*
    - Routine testing and status awareness
       *HIV testing, examinations, check-ups, testing frequency, preventive healthcare, awareness, education, sensitizing, increased awareness, better understanding*
    - Uptake of PrEP/ other biomedical prevention options
       *Injection drug use, drug abuse, drug addiction, unsafe sex, preventive measures, high-risk sexual behavior, HIV, human immunodeficiency virus, prescription, health, safety, quality of life*
    - Limiting sexual partners
       *Monogamy, intimate partner relations, safe sex, contraceptive use, abstinence, restraint, high-risk behavior, sexual behavior*
    - Raised age of sexual debut
  - Research engagement
    - Research awareness and benefit perception
       *Understanding, knowledge, education, inquiry, scientific inquiry, self-awareness, self-perception, perceptions, preconceived notions, involvement, engagement, values, interest, aid*
    - Participation in biomedical research
       *Community engagement, dialogues, discussions, awareness, knowledge building, community consultation, involvement, study, support, conversations, forums, meetings, learning, experiential learning, practical learning, practical training, practice, on-site training, in-field training, experience-based learning, community participation, community involvement, participatory practices, community contribution, participatory*
- Year of Publication
  - 2000-2004
  - 2005-2009
  - 2010-2014
  - 2015-2019
  - 2020-2021
- Funding Agency
  - 3ie
  - AusAid
  - BMGF
  - Canadian International Development Agency (CIDA)
  - Canadian Institutes for Health Research
  - Center for Disease Control and Prevention
  - Center for Interdisciplinary Research on AIDS (CIRA)
  - DFID UK
  - Duke Center for AIDS Research
  - Emory Center for AIDS Research
  - European Commission
  - ESRC, UK
  - Fogarty International Centre
  - Ford Foundation
  - Global Fund to Fight AIDS, Tuberculosis and Malaria
  - German Technical Corporation of DeutscheGesellschaft fuer Technische Zusammenerbeit (GTZ)
  - IAVI
  - IDRC, Canada
  - Irish Aid
  - National Institute on Alcohol Abuse and Alcoholism (NIAAA)
  - National Institute of Allergy and Infectious Diseases
  - National Institute of Child Health and Human Development
  - National Institute on Drug Abuse (NIDA)
  - NIH
  - NIMH
  - National Institute of Nursing Research
  - President's Emergency Plan for AIDS Relief
  - Rockefeller Foundation
  - Sigrid Rausing Trust
  - South African Medical Research Council
  - Stephen Lewis Foundation
  - Swedish International Development Cooperation Agency (SIDA)
  - UK Medical Research Council
  - UNAIDS
  - UNDP
  - UNFPA
  - UNICEF
  - USAID
  - Wellcome Trust
  - WHO
  - World AIDS Foundation
  - World Bank
  - Not Reported
  - Not received any funding
  - Funding Agency (other)
- AMSTAR-2
  - 1. Did the research questions and inclusion criteria for the review include the components of PICO?
    - Yes
    - No
  - 2*. Did the report of the review contain an explicit statement that the review methods were established before the conduct of the review and did the report justify any significant deviations from the protocol?
    - Yes
    - Partially Yes
    - No
  - 3. Did the review authors explain their selection of the study designs for inclusion in the review?
    - Yes
    - No
  - 4*. Did the review authors use a comprehensive literature search strategy?
    - Yes
    - Partially Yes
    - No
  - 5. Did the review authors perform study selection in duplicate?
    - Yes
    - No
  - 6. Did the review authors perform data extraction in duplicate?
    - Yes
    - No
  - 7*. Did the review authors provide a list of excluded studies and justify the exclusions?
    - Yes
    - Partially Yes
    - No
  - 8. Did the review authors describe the included studies in adequate detail?
    - Yes
    - Partially Yes
    - No
  - 9*. Did the review authors use a satisfactory technique for assessing the risk of bias (RoB) in individual studies that were included in the review?
    - Yes
    - Partially Yes
    - No
  - 10. Did the review authors report on the sources of funding for the studies included in the review?
    - Yes
    - No
  - 11*. If meta-analysis was performed, did the review authors use appropriate methods for statistical combination of results?
    - Yes
    - No
    - No meta-analysis was conducted
  - 12. If meta-analysis was performed, did the review authors assess the potential impact of RoB in individual studies on the results of the meta-analysis or other evidence synthesis?
    - Yes
    - No
    - No meta-analysis was conducted
  - 13*. Did the review authors account for RoB in primary studies when interpreting/discussing the results of the review?
    - Yes
    - No
  - 14. Did the review authors provide a satisfactory explanation for, and discussion of, any heterogeneity observed in the results of the review?
    - Yes
    - No
  - 15*. If they performed quantitative synthesis did the review authors carry out an adequate investigation of publication bias (small study bias) and discuss its likely impact on the results of the review?
    - Yes
    - No
    - No Meta-analysis was conducted
  - 16. Did the review authors report any potential sources of conflict of interest, including any funding they received for conducting the review?
    - Yes
    - No
  - Overall
    - High
       *No or one non-critical weakness*
    - Moderate
       *More than one non-critical weakness*
    - Low
       *One critical flaw with or without non-critical weaknesses*
    - Critically Low
       *More than one critical flaw with or without non-critical weaknesses*
- Study confidence
  - Low confidence SRs
  - Medium confidence SRs
  - High confidence SRs
  - Impact Evaluations

**Appendix F: Acronyms and Abbreviations**

| Acronym | Full Form | Page |
| --- | --- | --- |
| AGYW | Adolescent girls and young women | 1 |
| SBCC | Socio-behavioural change communication | 1 |
| LMIC | Low-and middle-income countries | 1 |
| EGM | Evidence and gap map | 1 |
| ICT | Information Communication and Technology | 1 |
| SRH | Sexual and reproductive health | 1 |
| HIV | Human immunodeficiency virus | 1 |
| STIs | Sexually Transmitted Infections | 3 |
| PWNM | Pregnant women and new mothers | 4 |
| PLHIV | People living with HIV | 4 |
| NACO | National AIDS Control Organization | 5 |
| WHO | World Health Organization | 5 |
| UNFPA | United Nations Population Fund | 5 |
| UNAIDS | The Joint United Nations Programme on HIV and AIDS | 5 |
| SDG | Sustainable Development Goals | 6 |
| NFHS | National Family Health Survey of India | 6 |
| SBC | Social and Behavioural Change | 7 |
| RMNCH | Reproductive, maternal, newborn and child health | 7 |
| HTC | HIV testing and counseling | 7 |
| VMMC | Voluntary medical male circumcision | 7 |
| PMTCT | Prevention of mother-to-child transmission | 7 |
| SRs | Systematic reviews | 8 |
| GNI | Gross National Income | 10 |
| TDF | Theoretical Domains Framework | 13 |
| COM-B Model | Capability, Opportunity, Motivation, and Behavior Model | 13 |
| PrEP | Pre-exposure prophylaxis | 15 |
| PEP | Post-exposure prophylaxis | 15 |
| ARV | Antiretroviral | 15 |
| RCT | Randomized Controlled Trial | 16 |
| IAS | International AIDS Society | 16 |
| HIVR4P | HIV Research for Prevention | 16 |
| JHU CPP | Johns Hopkins Center for Communication Programs | 16 |
| TISS | Tata Institute of Social Sciences | 16 |
| EGPAF | The Elizabeth Glaser Paediatric AIDS Foundation | 16 |
| URC | University Research Co. | 16 |
| PSI | Population Services International | 16 |
| ICRW | International Center for Research on Women | 16 |
| KHPT | Karnataka Health Promotion Trust | 16 |
| NACO-IBBS | National Integrated Biological and Behavioural Surveillance | 16 |
| HC3 | Health Communication Capacity Collaborative | 16 |
| 3ie | International Initiative for Impact Evaluation | 16 |
| GPP | Good Participatory Practices | 18 |
| ART | Antiretroviral therapy | 19 |
| PRISMA | Preferred Reporting Items for Systematic Reviews and Meta-Analyses | 23 |
| MENA | Middle East and North Africa | 30 |
